# Accurate inference of the full base-pairing structure of RNA by deep mutational scanning and covariation-induced deviation of activity

**DOI:** 10.1093/nar/gkz1192

**Published:** 2019-12-24

**Authors:** Zhe Zhang, Peng Xiong, Tongchuan Zhang, Junfeng Wang, Jian Zhan, Yaoqi Zhou

**Affiliations:** 1 High Magnetic Field Laboratory, Key Laboratory of High Magnetic Field and Ion Beam Physical Biology, Hefei Institutes of Physical Science, Chinese Academy of Sciences, Hefei 230031, Anhui, P. R. China; 2 University of Chinese Academy of Sciences, Beijing 101408, P. R. China; 3 Institute for Glycomics, Griffith University, Parklands Drive, Southport, QLD 4222, Australia; 4 Institute of Physical Science and Information Technology, Anhui University, Hefei 230031, Anhui, P. R. China; 5 School of Information and Communication Technology, Griffith University, Parklands Drive, Southport, QLD 4222, Australia

## Abstract

Despite the large number of noncoding RNAs in human genome and their roles in many diseases include cancer, we know very little about them due to lack of structural clues. The centerpiece of the structural clues is the full RNA base-pairing structure of secondary and tertiary contacts that can be precisely obtained only from costly and time-consuming 3D structure determination. Here, we performed deep mutational scanning of self-cleaving CPEB3 ribozyme by error-prone PCR and showed that a library of <5 × 10^4^ single-to-triple mutants is sufficient to infer 25 of 26 base pairs including non-nested, nonhelical, and noncanonical base pairs with both sensitivity and precision at 96%. Such accurate inference was further confirmed by a twister ribozyme at 100% precision with only noncanonical base pairs as false negatives. The performance was resulted from analyzing covariation-induced deviation of activity by utilizing both functional and nonfunctional variants for unsupervised classification, followed by Monte Carlo (MC) simulated annealing with mutation-derived scores. Highly accurate inference can also be obtained by combining MC with evolution/direct coupling analysis, R-scape or epistasis analysis. The results highlight the usefulness of deep mutational scanning for high-accuracy structural inference of self-cleaving ribozymes with implications for other structured RNAs that permit high-throughput functional selections.

## INTRODUCTION

The full base-pairing structure of RNA, resulted from the interplay of secondary and tertiary interactions, serves as a preformed frame for final folding of tertiary structure and is evolutionarily conserved to maintain the structural and functional integrity of an RNA (1). As such, it plays a prominent role in the versatility of RNA structure and function (2,3). To date, full base pairing structures at the single base-pair resolution can only be obtained from high-resolution RNA structures determined by X-ray crystallography, nuclear magnetic resonance or cryogenic electron microscopy. However, these traditional techniques solved only 4112 RNA structures as of 4 November 2018 (or 3% of all structures in protein databank (4) due to their requirement of nearly static structures that most RNAs do not have. Given that proteins are vastly outnumbered by noncoding RNAs (5) and the majority of these RNAs have unknown structures and functions, complementary alternative techniques for accurate determination of RNA base-pairing structures are urgently needed.

The most economical method for locating base pairs would be a computational prediction if its accuracy could be assured. Such a computational approach is usually referred to as RNA secondary structure prediction although many base pairs are associated with tertiary interactions (6). These tertiary contacts include noncanonical (non-Watson–Crick), non-nested (pseudoknot), and lone (single tertiary base pair not associated with a helix) base pairs. Despite the first secondary structure prediction method was developed nearly 50 years ago (7) and many advances have been made since (8,9), the problem remains unsolved: only 70% accuracy for predicted base pairs and 38% accuracy for secondary structure topology according to the most recent evaluation (10). In particular, lone and noncanonical base pairs are commonly ignored in secondary structure prediction, perhaps because they are not considered as a part of the secondary structure (6).

Large improvement in secondary structure prediction can be achieved (11,12) when computational algorithms are integrated with experimental restraints. These experimental results can be generated from structural probes such as enzymes, chemicals, hydroxyl radical, cross-linking, and mass spectrometry (13–15), in combination with next-generation sequencing (16). However, most of these experimental techniques measure one-dimensional reactivity profiles (17) and rely on computational approaches to infer two bases paired. As a result, they suffer the same limitations common for all existing computational methods: poor accuracy in locating lone, non-nested, and noncanonical base pairs (11,12,18).

Recently, more direct identification of base pairs can be made by multidimensional chemical mapping methods. Examples are mutate-and-map (M2 (19)), SHAPE and mutational profiling (SHAPE-MaP (20)), RNA interaction groups by mutational profiling (RING-MaP (21)), multiplexed ⋅OH cleavage analysis (MOHCA (22)), and modify-cross-link-map (MXM (23)). While these multidimensional chemical mapping methods can detect RNA base pairs at the helix level (>2 bp) (24), they are not yet sensitive enough to detect all base pairs individually, lone and two contiguous base pairs, in particular. This is in part because the assumption of a localized response upon perturbation is not always true (23). Moreover, these experiments are labor intensive and require sophisticated computationally-intensive algorithms for data analysis (23,25).

Another powerful technique is deep mutational scanning through function selection in combination with high-throughput sequencing (26–33). However, current analysis of deep mutation data has been limited to mapping functional fitness landscape (26,28–30,32) or locating helices via covariation analysis (27,31–33). Separately, sophisticated statistical covariation analysis such as R-scape, evolutionary coupling, or direct coupling has been successfully applied to naturally occurring mutant variants within a large RNA family (34–36). However, this analysis requires a large number of known homologous sequences within the same functional family, of which the majority of noncoding RNAs do not have. Moreover, the accuracy of such analysis strongly depends on the quality and quantity of homologous sequences.

Unlike naturally occurring homologous sequences, the quality and quantity of functional mutants can be somewhat controlled in deep mutational scanning. Thus, it is highly desirable if deep mutation data alone can be used to reliably infer complex secondary and tertiary base pairing structures including lone base pairs not detectable by current chemical probing techniques. We demonstrate the feasibility by employing self-cleaving ribozymes that catalyze their own cleavage. These ribozymes are particularly suitable for deep mutational scanning (29) because the functional activity of a mutant can be calculated by simply counting the number of cleaved and uncleaved reads of that mutant in high-throughput sequencing data. More importantly, self-cleaving ribozymes have complex base-pairing structures, each of which has its own interesting mechanistic difference (37–39). Thus, they offer an ideal testing ground for inferring different base pairing topologies from deep mutational scanning. Moreover, self-cleaving ribozymes are broadly distributed in genomes of different organisms from viroids to vertebrates (40–42). In human genome alone, we have seven known self-cleavage ribozymes (CPEB3, LINE1, OR4K15, IGF1R, HH9, HH10 and CoTC ribozymes) (43–45) and none of them has structure determined. This highlights the importance of solving structures of self-cleavage ribozymes even if at the level of base pairing.

**Figure 1.**
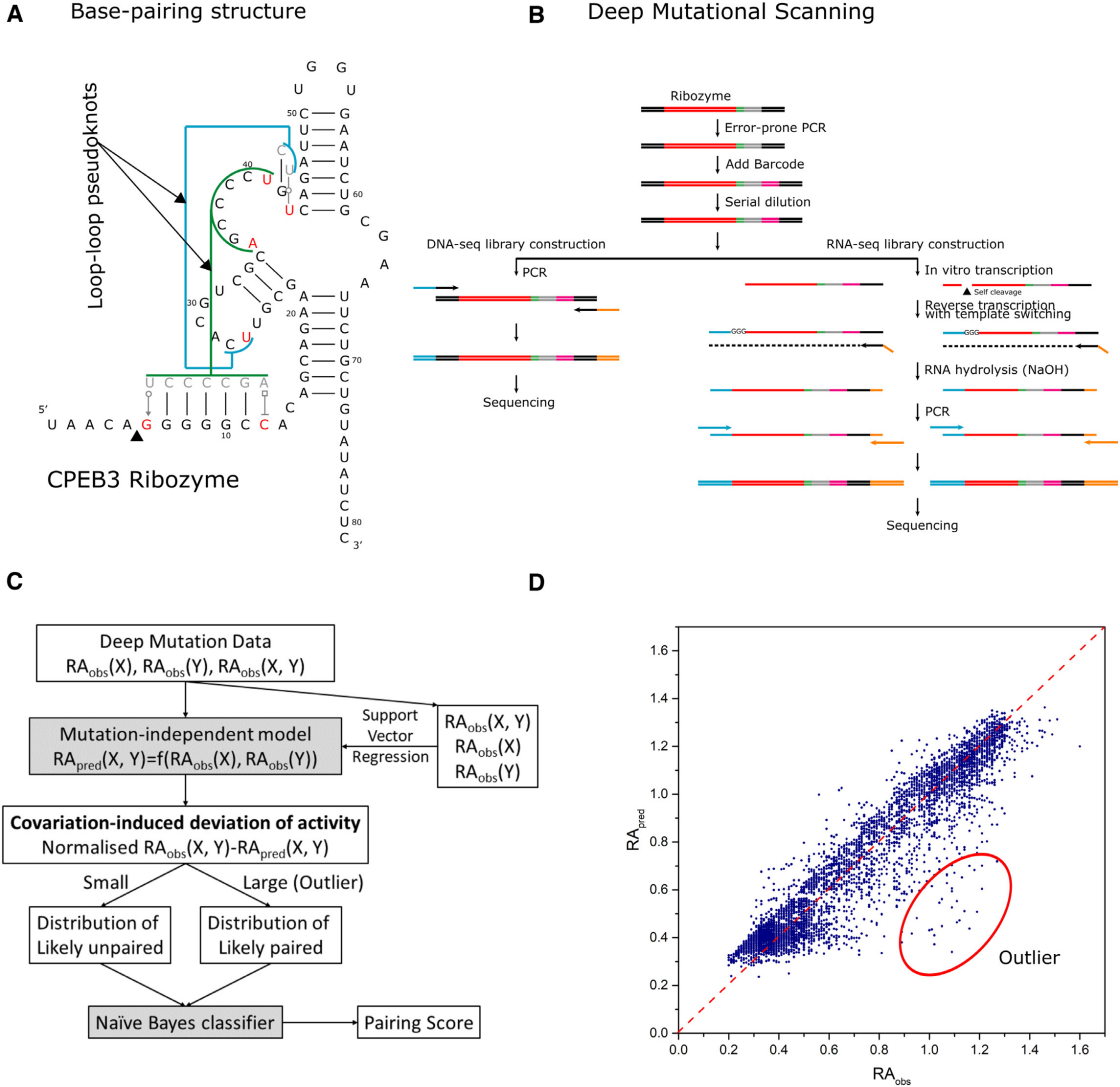
(**A**) The native base-pairing structure of the 81-nucleotide CPEB3 ribozyme contains three canonical helical regions. In addition, it has one pseudoknot capped by two non-Watson–Crick (WC) base pairs (G6U41 and C12A35) and another pseudoknot made of a lone WC pair and one non-WC pair (U26U43). (**B**) The deep mutational sequencing of CPEB3 ribozyme starts from random mutagenesis via error-prone PCR, followed by barcoding so that the whole ribozyme sequences in DNA-seq can be mapped onto self-cleaving reaction products from the RNA-seq library. (**C**) Analyzing deep mutation data by covariation-induced deviation of activity. This is done by constructing an independent-mutation model and building a naïve Bayes classifier for base pairs according to distributions of unlikely paired bases and likely paired bases (outliers of the independent-mutation model). (**D**) The activities of double mutants observed from sequencing versus those predicted by the independent- mutation model. Outliers for likely base pairs are indicated by the red circle.

Here, we performed deep mutational scanning of the self-cleaving 81-nucleotide CPEB3 ribozyme located at the intron region of the human gene of cytoplasmic polyadenylation element–binding protein 3 (CPEB3). Although the tertiary structure of CPEB3 ribozyme remains to be solved, it will fold into a hepatitis delta virus-like base pair pattern confirmed by mutational studies (43). This ribozyme was chosen for its complex base-pairing structure. One pseudoknot is non-helical, made of one lone canonical Watson–Crick (WC) pair and one noncanonical pair and the other is capped by two noncanonical base pairs, all associated with tertiary interactions (Figure 1A). Moreover, this ribozyme is of biological significance because a single nucleotide polymorphism was found to affect CPEB3 ribozyme activity with a difference in episodic memory (46). We established a method that analyzes covariation-induced deviation of activity (CODA) by using Support Vector Regression (SVR) to establish an independent-mutation model and a naïve Bayes classifier to separate bases paired from unpaired. This unsupervised CODA analysis improves the signal-to-noise ratio by employing both functional and nonfunctional variants. Moreover, incorporating Monte-Carlo simulated annealing with a commonly-used energetic model and a CODA scoring term further improves the coverage of the regions under-sampled by deep mutations. Accurate determination of secondary and tertiary base pairs is further confirmed by CODA-based analysis of the available deep mutation data of the downstream portion of the cleavage site of a twister ribozyme (a 48-nucleotide fragment of 54 nucleotide *Oryza sativa* Osa-1–4 ribozyme sequence) (29).

## MATERIALS AND METHODS

### Deep mutational scanning of self-cleaving CPEB3 ribozyme

The overall procedure for deep mutational scanning of CPEB3 ribozyme is shown in Figure 1B. All oligonucleotides were purchased from IDT. The sequences of oligonucleotides used in this experiment are listed in Supplementary Table S1.

Firstly, a mutation library was constructed by randomly mutating the wild type sequence of CPEB3 ribozyme with several rounds of epPCR (47) using primer T7prom and M13F (Supplementary Table S1). The amplification product of each round was purified, quantified, and diluted for use as the DNA template in the next round of epPCR. PCR products after several rounds of epPCR were barcoded using primer Bar_F and Bar_R (Supplementary Table S1) by a low-cycle PCR (cycle number = 3). The purified barcoding product was then loaded to a denaturing 8% PAGE with 8 M urea for separation. The target band containing barcoded DNAs was excised and recovered by passive elution in crush-soak buffer (10 mM Tris–HCl, pH 7.5, 200 mM NaCl, 5 mM EDTA) overnight at 37°C followed by ethanol precipitation. The recovered DNAs were dissolved in water and then quantified by absorbance and qPCR. Approximately 10^5^ barcoded DNA molecules were amplified by T7prom and M13F primers (Supplementary Table S1) in order to produce enough DNAs for the downstream steps.

Then, the mutant DNA library was transcribed *in vitro* in a 30 μl reaction system containing 5 pmol of the dsDNA template, 2 mM NTPs, 1× RNAPol Reaction Buffer (New England Biolabs), 1 U/μl Murine RNase Inhibitor (New England Biolabs), and 5 U/μl T7 RNA polymerase (New England Biolabs) for 5 hours at 37°C. Template DNA was removed by adding 58 μl nuclease-free water, 10 μl of 10× DNase I Buffer (New England Biolabs), and 2 μl of DNase I (2 U/μl, New England Biolabs) to 30 μl RNA product, and incubated at 37°C for 15 min. The active ribozyme mutants were simultaneously cleaved during *in vitro* transcription and DNase I treatment. The buffer condition was not optimized as we employed the relative activity instead of the cleavage ratio in our analysis. The relative activity over wild type is much less sensitive to different experimental conditions than the cleavage ratio. 2 μl of 0.5 M EDTA (to a final concentration of 10 mM) was added to stop both ribozyme and DNase I activities. The transcribed RNAs were purified using RNA Clean & Concentrator-5 kit (Zymo Research) and quantified by absorbance.

Afterward, ∼10 pmol purified RNAs were mixed with 2 μl of 10μM RT_m13f_adp1 (Supplementary Table S1) and 1 μl of 10mM dNTP in a volume of 8 μl and heated to 65°C for 5 min and placed on ice. Reverse transcription was initiated by adding 4 μl of 5× ProtoScript II Buffer (New England Biolabs), 2 μl of 0.1 M DTT, 0.2 μl of Murine RNase Inhibitor (40 U/μl, New England Biolabs), 1 μl ProtoScript II RT (200 U/μl, New England Biolabs), and 2 μl 100μM template-switching oligonucleotide TSO (Supplementary Table S1) to a total volume of 20 μl. The reaction mixture was incubated at 42°C for 1 h, then inactivated at 80°C for 5 min. RNA was removed by adding 1 μl 5M NaOH and heating at 95°C for 5 min.

Next, DNA-seq libraries were constructed by extending the ribozyme mutant library with P5R1_m13f and P7R2_t7p (Supplementary Table S1) in the touch-up PCR reaction (2 cycles of PCR with the annealing temperature at 53°C, followed by 8 cycles of PCR with the annealing temperature at 67°C). Similarly, the RNA-seq libraries were constructed by extending the purified cDNA after template switching reaction with P5R1_adp1 and P7R2_adp2 (Supplementary Table S1).

Lastly, the DNA-seq and RNA-seq libraries were sequenced on an Illumina HiSeq X sequencer with 25% PhiX control by Novogene Technology Co., Ltd. The reads were first assessed using FastQC (https://www.bioinformatics.babraham.ac.uk/projects/fastqc/). Raw paired-end reads were merged using program SeqPrep (John, J.S., 2011. https://github.com/jstjohn) to generate high-quality full-length reads. For DNA-seq results, reads with a correct match to primers Bar_F and Bar_R were extracted to generate the projection map from barcodes to variants. For multiple variant sequences mapped to one barcode, the partial sequence downstream to the cleavage site was employed as the additional marker for tracking different mutants. If multiple mapping was still an issue, only the consensus sequence with the frequency larger than 50% was used. For RNA-seq results, reads with a correct match to primer Bar_R were extracted. The barcode together with partial sequences downstream to the cleavage site was used to map the full-length ribozyme sequence. Then, we counted the cleaved and uncleaved read numbers for each variant to calculate relative activity (RA), using the equation given by RA(var) = *N*_cleaved_(var)*N*_total_(wt)/*N*_total_(var)*N*_cleaved_(wt). To compute RA, we set the minimum number of reads to 5.

We performed three batches of experiments. They differ in the number of rounds in epPCR (3, 8 and 5 rounds for Batches 1, 2, 3, respectively). Moreover, in Batch 2, the cDNA with template switching was used to construct the RNA-seq library directly. In Batches 1 and 3, the cleaved and uncleaved cDNAs were separated by PAGE for RNA-seq library construction and then mixed with a near equal molar ratio for sequencing. The purpose was to improve the sampling of functional sequences. The DNA and RNA sequencing results, the number of mutations, mutation rates, and the median and maximal reads per variant in CPEB3 ribozyme for these batches were summarized in Supplementary Tables S2–S5, respectively. Supplementary Figure S1 also showed position-dependent mutation rates of three batches. To obtain a higher coverage of mutation variants, we merged the RA results together. Average RA was used if a variant appeared in different batches. Because the coverage for double mutations in CPEB3 ribozyme was low (41.0%), we increased the coverage by including triple mutants for those position pairs without double mutations in our CODA analysis as follows. For a triple mutant XYZ, we calculate RA(XY) = RA(XYZ)/RA(Z) when RA(Z) is larger than 0.5. Here, we have assumed that Z mutation does not covariate with XY mutations. In this way, these triple mutants were treated the same as double mutants in CODA analysis. This extension increased the double mutation coverage from 41.0% to 61.3% (Supplementary Table S6). For twister ribozyme, triple mutants are not included because of 100% coverage of double mutations.

### Covariation-induced deviation of activity (CODA)

The overview of the method is shown in Figure 1C. Specific details are described below.

#### The independent-mutation model

The basic assumption of CODA analysis is that the effect of two mutations on enzymatic activity is independent if they are not in close contact. This assumption is an approximation in the case that long-range interactions or allosteric effects are important. Under this approximation, the RA of double mutants can be modeled (predicted) by the RAs of two single mutants. However, if two bases form a base pair, the RA of their double mutation to another complementary pair will be higher than two independent single mutations that are disruptive to the wild-type pair. In other words, we can detect base pairs by examining Covariation-induced Deviation of the Activity (CODA) of a double mutant from the independent-mutation model. We constructed this model by using an SVR regression of the RA of all double-mutation variants against RAs of their corresponding single-mutation variants. This was done by organizing RA data into a three-dimensional vector of RA_obs_(bi), RA_obs_ (bj), and RA_obs_(bi, bj), which are RAs of two single mutation variants and the double mutation variant, respectively. Then, these data points are used to generate the SVR model to fit RA_obs_(bi, bj) as a function of RA_obs_ (bi) and RA_obs_ (bj). This SVR model is used to predict the activity for any double mutations as we have 100% coverage for single mutations of twister and CPEB3 ribozymes. Comparison between observed and predicted *RA* values is shown in Figure 1D for CPEB3 ribozyme and Supplementary Figure S2 for a twister ribozyme. Our nonlinear SVR regression model was generated using the python package scikit-learn with a radial basis function kernel $K\ ( {{\boldsymbol{x}},{\boldsymbol{x^{\prime}}}} ) = \ {\rm{exp}}( - \gamma {| {| {{\boldsymbol{x}} - {\boldsymbol{x^{\prime}}}} |} |^2})$ where ${\boldsymbol{x}}$ and ${\boldsymbol{x^{\prime}}}$ are two feature vectors. In addition to the γ parameter, it has a hyperparameter C to control the margin of separating hyperplanes. We employed C = 2 × 10^3^ and γ = 2.0 to optimize the regression. We found that C from 1 to 10^4^, and γ from 0.01 to 10 did not change the results (Supplementary Table S7). We emphasize that the model is based on deep mutation data only without requiring the knowledge of base pairs.

#### CODA calculation

The CODA score is defined as the difference between the actual RA value (RA_obs_) and the predicted RA (RA_pred_) from the above regression model normalized by a shifted predicted *RA* value. CODA = (RA_obs_ – RA_pred_)/(RA_pred_ + 0.2). A shift (0.2) is used to avoid the small value of RA_pred__._ The results do not depend on this value from 0.1 to 0.5. Here, dividing by RA_pred_ is to obtain the relative change in activity.

#### CODA distribution

We expected that the distribution of CODA scores for double mutations is a mixture of two distributions approximated as Gaussian: one centered near 0 for structurally unpaired bases (two mutations are independent) and one centered at a higher value for potential base pairs as illustrated in Figure 1D and Supplementary Figure S2. However, the dominance of non-paired over paired bases makes it challenging to de-mix the two distributions. Thus, we employed the following empirical method. The average (*a*_1_) and standard deviation (*sd*_1_) of the first distribution were obtained from all double mutation data, as the effect of the second distribution is negligible. Then, the average (*a*_2_) and standard deviation (*sd*_2_) of the second distribution were obtained by the subset of data points with a CODA score > *a*_1_ + 3*sd*_1_. This subset also defines the overall base-pair probability (P(paired)) calculated as the portion of the subset in all double mutations. Varying this 3*sd*_1_ cut off from 1 *sd*_1_ to 7 *sd*_1_ makes minor changes in distributions and the final result (Supplementary Table S7).

For CPEB3 ribozyme, the distributions for paired and unpaired bases obtained by the above method have mean values at 1.26 and –0.004 and deviations at 0.763 and 0.237, respectively, with the overall base-pair probability (P(paired)) at 0.013. For twister ribozyme, the distributions for paired and unpaired have mean values at 1.71 and –0.116 and deviation at 1.111 and 0.222, respectively, with the overall pair probability *P*(paired) = –0.079. Again, there is no specific assumption about which two bases are paired or not paired.

#### Pairing score by the Bayes classifier

The pairing score of a double mutation (Ps) is calculated by a naïve Bayes two-state classifier with

$$\begin{equation*}{\rm Ps}\ = \log\frac{{p\left( {{\rm CODA}{\rm{|}}{\rm paired}} \right)}}{{p\left( {{\rm CODA}} \right)}}\end{equation*}$$

with $p\ ( {{\rm CODA}} ) = \ p( {{\rm CODA}{\rm{|}}{\rm paired}} )*p( {{\rm paired}} ) + p( {{\rm CODA}{\rm{|}}{\rm unpaired}} )*( {1 - p( {{\rm paired}} )} )$. The probability is derived from the Mixture Gaussian model, without using a training set as described above.

### Secondary structure prediction by Monte Carlo simulated annealing

We built a simple secondary structure predictor to examine if it can be used to fill the gap in the region of contiguous base pairs with weak covariation signals. We defined the energy score as follows.

$$\begin{eqnarray*} E &=& \Delta {G_{37\ {\rm stacking}}} + \Delta {G_{37\ {\rm AU}\ {\rm end}\ {\rm penalty}}}\left( {{\rm per}\ {\rm AU}\ {\rm end}} \right)\\ &&+\, \Delta {G_{{\rm lone}}} + \Delta {G_{{\rm non} - {\rm AU}/{\rm GC}/{\rm GU}}}\end{eqnarray*}$$

where the stacking energy Δ${G_{37\ {\rm stacking}}}$ and the penalty of helical AU end Δ${G_{37\ {\rm AU}\ {\rm end}\ {\rm penalty}}}( {{\rm per}\ {\rm AU}\ {\rm end}} )$ were obtained from Tuner 2004 experimental data (48). Δ${G_{{\rm lone}}}$ and Δ${G_{{\rm non} - {\rm AU}/{\rm GC}/{\rm GU}}}$are the penalties for a lone pair and for non-AU, GC or GU (wobble) pairs, respectively. To make the simplest model, we used a single value for both Δ${G_{{\rm lone}}}$ and Δ${G_{{\rm non} - {\rm AU}/{\rm GC}/{\rm GU}}}$penalties. We found the low penalty value by trials and errors to ensure this simple secondary structure prediction not to yield any lone or non-AU/GC/GU pair. In each Monte Carlo step, a random existing pair was destroyed, or a new pair was generated. The move is accepted according to the Metropolis criterion. At each temperature, we performed 500 000 Metropolis steps. The initial temperature was set at 10 and decreased by a factor of 0.95 at each round until temperature reached 0.1. The results were stable when the single adjustable penalty parameter varied between 2 and 5 kcal/mol.

After the above energy function was defined, we carried out the CODA-guided base-pairing prediction by using *E* – W*Ps/*mean* with an optimized weight *W*, relative to the mean of pairing scores. The simulated annealing procedure was the same as above for using MC alone.

### Covariation analysis by mutational coupling analysis

#### Defining functional sequences for mutational coupling analysis by an RA cutoff

Functional variants with high ribozyme activity were used as the input sequences for covariation analysis by R-scape, EC-RNA and mfDCA-RNA. We have tested different RA cutoffs (0.0, 0.1, 0.2, …, 0.9, 1) for defining functional sequences (1 can be used because many sequences have stronger activities than the wild type). The result was not sensitive to RA between 0.5 and 0.7 (See Supplementary Figure S3). As a result, we mainly reported the result with RA cut off at 0.5 (Table 1).

**Table 1.** Performance of R-scape, mean-field direct coupling analysis (mfDCA-RNA), evolutionary couplings (EC-RNA), covariation-induced deviation of activity (CODA, this work), Monte Carlo simulated annealing (MC), R-scape+MC, mfDCA-RNA+MC, EC-RNA+MC and CODA+MC for inferring base pairs from deep mutation data in term of area under the precision-recall curve (AUC_PR), Matthews correlation coefficient (MCC), sensitivity, and precision for CPEB3 and twister ribozymes. Results for RNA secondary structure predictors (RNAfold and IPknot) are also shown for comparison

Method	AUC_PR	MCC	Sensitivity	Precision
CPEB3 (81 bases, 26 base pairs)				
RNAfold	-	0.66	0.65	0.68
IPknot	-	0.71	0.73	0.70
R-scape	0.30	0.42	0.35	0.53
mfDCA-RNA	0.48	0.59	0.35	1.00
EC-RNA	0.44	0.59	0.38	0.91
CODA	0.53	0.62	0.38	1.00
MC	0.48	0.60	0.77	0.48
R-scape+MC	0.92	0.92	0.92	0.92
mfDCA-RNA+MC	0.88	0.90	0.88	0.92
EC-RNA+MC	0.95	0.93	0.96	0.89
CODA+MC	0.97	0.96	0.96	0.96
Twister fragment (48 bases, 17 bp)				
RNAfold	-	0.22 (0.68 a)	0.18 (0.47^a^)	0.30 (1^a^)
IPknot	-	0.0 (0.68 a)	0.0 (0.47^a^)	0.0(1^a^)
R-scape	0.54	0.64	0.47	0.89
mfDCA-RNA	0.66	0.66	0.71	0.63
EC-RNA	0.59	0.66	0.71	0.63
CODA	0.70	0.79	0.76	0.81
MC	0.58	0.67	0.59	0.77
R-scape+MC	0.84	0.84	0.76	0.93
mfDCA-RNA+MC	0.90	0.91	0.88	0.94
EC-RNA+MC	0.89	0.88	0.82	0.93
CODA+MC	0.91	0.91	0.82	1.00

a Folded using the whole sequence (54 bases), rather than the 48-base fragment.

#### R-scape

R-scape was downloaded from http://eddylab.org/R-scape/. Default parameters except *E*-value were used in this paper. We changed the *E*-value significance threshold from 0.05 to 1000 in order to achieve the best performance for our datasets. We assigned a score of 0 to base pairs which did not have a score in R-scape result.

#### Direct/evolution coupling analysis

There are many programs for mutational direct coupling analysis. Here we only employed two programs (EC-RNA and mfDCA-RNA, respectively) applied to RNA previously (35,36). For EC, we followed the pipeline https://github.com/debbiemarkslab/plmc. Due to the high sequence identity of our mutation library, we remove the sample reweighting process by modifying the reweighting parameter theta to 0. The maximum number of iterations was changed from default value 50–500 in our paper, to achieve better performance. mfDCA-RNA was downloaded from http://dca.rice.edu/portal/dca/home. As in EC-RNA, we changed the sequence reweighting factor theta from 0.2 to 0. Average product correction to the final mfDCA score was not employed because it decreases the performance for inferring from mutational libraries.

#### Epistasis

The fitness of a ribozyme variant was calculated as the natural logarithm of the relative activity of the variant. Then epistasis was calculated from a non-parametric null model following Schmiedel & Lehner (Epi-SL) (49). The combined score from the three interaction scores was used to estimate the epistatic interactions for our ribozymes. We also directly obtained the result from epistasis analysis of twister by Rollins *et al.* (Epi-Rollins) (50).

### Performance measurement

For binary classification of bases paired and unpaired, we measured the performance of different methods by Matthews correlation coefficient (MCC), sensitivity (recall), and precision. MCC, sensitivity, and precision are defined by

$$\begin{equation*}{\rm MCC}{\rm{\ }} = {\rm{\ }}\frac{{{\rm TP} \times {\rm TN} - {\rm FP} \times {\rm FN}}}{{\sqrt {\left( {{\rm TP} + {\rm FP}} \right) \times \left( {{\rm TP} + {\rm FN}} \right) \times \left( {{\rm TN} + {\rm FP}} \right) \times \left( {{\rm TN} + {\rm FN}} \right)} }},\end{equation*}$$

$$\begin{equation*}{\rm{Sensitivity\ }} = {\rm{\ }}\frac{{{\rm TP}}}{{\left( {{\rm TP} + {\rm FN}} \right)}},\end{equation*}$$

$$\begin{equation*}{\rm{Precision\ }} = {\rm{\ }}\frac{{{\rm TP}}}{{\left( {{\rm TP} + {\rm FP}} \right)}}\end{equation*}$$

where TP, TN, FP and FN represent True Positive (base pairs), True Negative (two bases unpaired), False Positive (two bases unpaired but predicted as paired) and False Negative (two bases paired but predicted as unpaired), respectively. Sensitivity (or recall) is a measure of the fraction of correctly predicted base pairs in all known base pairs (i.e. the percent of the coverage of all native base pairs). Precision is a measure of the fraction of correctly predicted base pairs in all predicted base pairs. RNA bases unpaired are about 50 times more than base pairs. For such an unbalanced system, MCC is a balanced measure (51). MCC is determined by a threshold that separates paired from unpaired. The maximum value of MCC along with sensitivity and precision at the same cut off are reported here for the comparison between different methods. We also employed the precision-recall curve rather than the receiver operator characteristic curve because our main interest is the minor class of positive samples (51) (in this case, base pairs).

### RNAfold and IPknot

Secondary structure predictions using RNAfold and IPknot were calculated through their web servers at http://rna.tbi.univie.ac.at/cgi-bin/RNAWebSuite/RNAfold.cgi and https://rtips.dna.bio.keio.ac.jp/ipknot/, respectively.

### Self-cleaving twister ribozyme

Twister ribozyme data and processing perl scripts were provided by Dr Yokobayashi (29). 43 229 917 uncleaved and 15 008 244 cleaved reads were extracted from the raw data. Relative activity of each variant was calculated by the following equation: RA(var) = *N*_cleaved_(var)*N*_total_(wt)/*N*_total_(var)*N*_cleaved_(wt).

### Fluorescence-based kinetic analysis

Firstly a ribozyme sequence was separated into two parts: the substrate part (CP_S, Supplementary Table S1) and the enzymatic part (CP_E, Supplementary Table S1). CP_S was synthesized and HPLC purified by IDT with 5′ 6-FAM as fluorophore and 3′ TAMRA as quencher. Cleavage of CP_S by CP_E will relieve the quenching and therefore generate a fluorescence signal. The T7 transcription cassette of CP_E was constructed by PCR, digested by BspQI to make a defined 3′ end, and then transcribed *in vitro* by T7 RNA transcription system as described before. For one 100 μl reaction system, 50 pmol purified CP_E was mixed with 10 pmol CP_S. Prior to the cleavage reactions, the mixed RNAs were denatured at 85°C for 5 min, then annealed in 0.5 mM Tris, 0.05 mM EDTA at pH 7.5 and 37°C for 10 min. Prewarmed 10× RNAPol Reaction Buffer (New England Biolabs) and H_2_O were added to the annealed RNAs to start the reaction. The fluorescence (Ex 488 nm/Em 520 nm) was measured at 37°C for 6h using TECAN infinite M200 PRO. First-order rate constant of ribozyme cleavage *k*_cat_ was calculated in a similar way as PAGE-based assay (52). The kinetics data were fitted to$\ F\ = \ A - B{{\rm e}^{ - {k_{{\rm cat}}}t}}$, where *F* is the fluorescence intensity, *t* is time, *A* is the fluorescence at completion, and *B* is the amplitude of the observable phase. Each data point in Supplementary Figure S5 is the average of *k*_cat_ of 4 independent reaction systems.

## RESULTS

### Deep mutational scanning experiment

The deep mutational scanning experiment of CPEB3 ribozyme was carried out by random mutations generated from error-prone PCR (epPCR) as shown in Figure 1B with mutation rates varied from 1.73% to 5.62% in three different batches so as to maximize the coverage of double and triple mutations. The resulting mutation library was barcoded and separated for DNA sequencing (DNA-seq) and *in vitro* transcription to RNAs, respectively. Transcribed RNAs self-cleaved during the transcription process. These self-cleaved and non-cleaved RNAs were then reverse-transcribed back to DNA for sequencing (RNA-seq). DNA-seq was used to obtain the full sequences of mutation variants that can be mapped to the reaction products from RNA-seq according to barcodes. This mapping was necessary to calculate the relative activity of each variant based on the fraction of cleaved fragments of the variant from RNA-seq, relative to that of the wild-type sequence. Combining three batches of experiments led to the deep mutation data of CPEB3 ribozyme with a total of 243 single, 11 968 double, 36 214 triple and 62 992 other (>3) mutants, along with measured enzymatic activities. This represents 100% coverage of single mutations but only 41.0% coverage of all possible double mutations. The low coverage of double mutations is in part because error-prone PCR tends to have fewer mutations in the GC than in the AT region (47,53). To expand the coverage of double mutations, we approximated triple as double mutations for those position pairs not covered by double mutations (see Methods) and improved the coverage to 61.3% of possible double mutants with 86.9% for AT and 31.0% for GC pairs. The low mutation coverage for GC pairs and the small library (<50 000 single-to-triple mutation variants) make it challenging for covariation analysis.

### Confirmation of functional activity measured from deep mutational scanning

To confirm functional activities of mutants measured from deep mutational scanning, we chose the wild type ribozyme and 11 mutants with varied relative activities in deep mutational scanning, and measured their *k*_cat_ by a fluorescence assay (Supplementary Figure S4). As shown in Supplementary Figure S5, the measured activities by the fluorescence assay are highly consistent with those from deep mutational scanning with 0.914 for the Pearson correlations. This confirms the quality of functional activities generated from high-throughput techniques.

### Covariation-induced deviation of activity (CODA)

The deep mutation data obtained above was employed to search for the base pairs whose covariation (co-mutation) would lead to the recovery of cleavage activity, disrupted by single mutations. To build a sensitive method, we first employed support vector regression to establish an independent-mutation model in which the relative activity of a double mutant is predicted by the activities of two corresponding single mutants (Figure 1C). Then, the deviation of the observed activity from the predicted activity measures the strength of covariation, with outliers associated with potential base pairs (Figure 1D). The distribution of these covariation-induced deviations of activity (CODA) can be deconvoluted into two separate distributions for independent double mutations centered at CODA = 0 and outlier double mutations for likely base pairs, respectively (Figure 1C, see Materials and Methods). A naïve Bayes classifier was obtained to estimate the probability of two bases paired. Both the SVR model and the Bayes classifier were obtained from the deep mutation data alone without making any explicit/implicit assumptions about which two bases are paired or not paired (i.e. unsupervised). The performance is robust against a few parameters employed for unsupervised clustering (see Methods and below). In other words, it is a predictive model applicable to RNAs with unknown base-pairing structures.

### Performance

Table 1 compares the performance of CODA on inferring true base pairs from the deep mutation data of CPEB3 ribozyme with R-scape, evolutionary couplings (EC-RNA) and mean-field direct coupling analysis (mfDCA-RNA) that have already been successfully applied to extract contacts from RNA families (34–36). To enhance the performance of EC-RNA and mfDCA-RNA, we increased the maximum number of iterations from 50 to 500 in EC-RNA and removed the reweighting scheme for sequences with different levels of sequence identities to the wild type because our sequences are all highly homologous to each other. For R-scape, *E*-value was changed to 1000 to optimize its performance. Unlike CODA, all mutants regardless of the number of mutations are employed in R-scape, mfDCA-RNA and EC-RNA. There are many other direct coupling analysis techniques for inferring protein contact maps (e.g. gplmDCA (54), PSICOV (55) and plmDCA20 (54)). We do not compare them here because optimizing their parameters for RNA is out of the scope of the current study. Here, for method comparison, the thresholds for defining base pairs were all chosen to maximize the Matthews correlation coefficient (MCC) value. As Table 1 shows, EC-RNA and mfDCA-RNA have the same performance in term of Matthews correlation coefficients (MCC = 0.59, note that MCC is 1 for perfect prediction and 0 for random prediction) and mfDCA-RNA has a slightly higher area under the precision–recall curve (AUC-PR = 0.48, 0.44, respectively), while R-scape has a moderate performance in term of MCC (MCC = 0.42) and AUC_PR (AUC_PR = 0.30). CODA provides 10% improvement in AUC-PR and 5% improvement in MCC over mfDCA-RNA.

**Figure 2.**
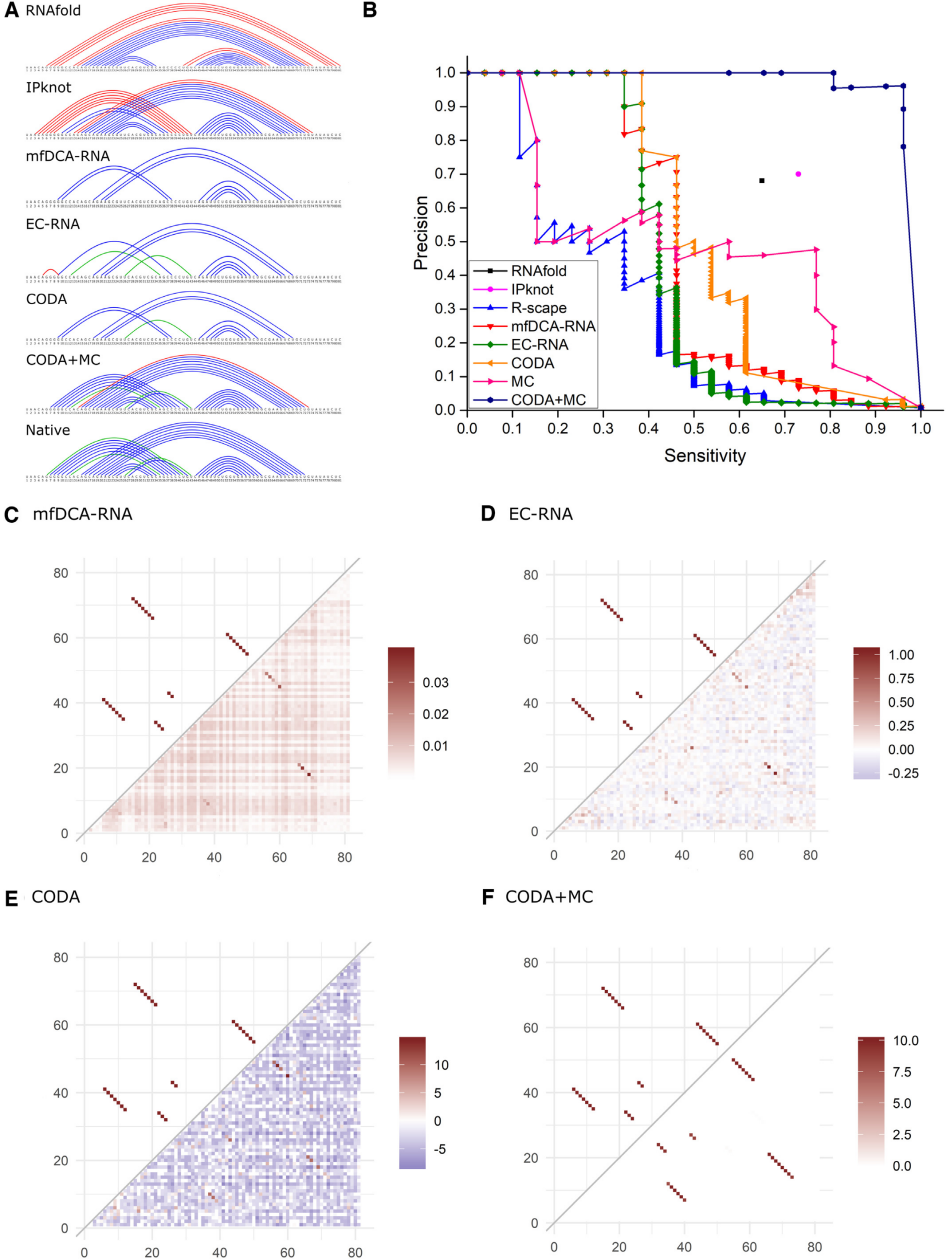
(**A**) The native base pairs of the CPEB3 ribozyme along with base pairs predicted by secondary structure predictors RNAfold and IPknot and inferred from its deep mutation data by mean-field direct coupling analysis (mfDCA-RNA), evolutionary couplings (EC-RNA), covariation-induced deviation of activity (CODA), and CODA integrated with a Monte Carlo simulated annealing (CODA+MC). Native Watson–Crick and non-Watson–Crick base pairs are shown in blue and green, respectively. False positive predictions are shown in red. (**B**) Precision (fraction of correct base pairs in predicted base pairs) versus sensitivity (coverage of known base pairs) by R-scape, mfDCA-RNA, EC-RNA, CODA, MC and CODA+MC, using the deep mutation data from CPEB3 ribozyme. Results from secondary structure predictor (RNAfold and IPknot) are also shown as points. (**C**) The comparison between native base-pairing map (upper triangle) of CPEB3 ribozyme and the map inferred from CPEB3 ribozyme deep mutation data (lower triangle) by mfDCA-RNA. (**D**), (**E**) and (**F**) are the same as (C) but for EC-RNA (D), CODA (E) and CODA+MC (F), respectively.

Figure 2A compares the actual secondary structure given by various methods. Although Table 1 indicates that the overall performance of mfDCA-RNA, EC-RNA, and CODA is lower than secondary structure predictors (either RNAfold (MCC = 0.66) or IPknot (MCC = 0.71)). However, RNAfold can only predict nested helical stems. IPknot can predict the pseudoknot with the long contiguous region of stacked base pairs but not the short, nonhelical one. By comparison, EC-RNA (but not mfDCA-RNA) and CODA can capture some nested along with some non-nested base pairs from both pseudoknots as shown in Figure 2A.

Figure 2B compares precision as a function of sensitivity or precision-recall curves given by various methods. Systematic improvement of CODA over mfDCA-RNA and EC-RNA is clearly demonstrated with 77%, 20% and 10% increase, respectively, in AUC_PR over R-scape, EC-RNA and mfDCA-RNA, respectively. At the maximal MCC value, EC-RNA yields 10 correct base pairs and 1 false positive prediction and mfDCA-RNA has nine correct predictions, compared to 10 correct base pairs at 100% precision by CODA (Figure 2A), while R-scape has nine correct base pairs and eight false positives (Supplementary Figure S6). It is particularly encouraging to note that R-scape, EC-RNA and CODA can capture one (UU) or two (UU and GU) out of three noncanonical pairs. Neither RNAfold nor IPknot can predict any noncanonical pairs as these base pairs are commonly ignored in secondary structure prediction. This confirms the usefulness of deep mutational scanning for revealing high-quality correlated mutations in tertiary contacts.

What makes CODA a better performer in detecting base pairs? Figure 2C–E shows the density plot for base-pairing maps given by mfDCA-RNA, EC-RNA and CODA, respectively. The predicted scores for all base pairs by CODA have the widest distribution (from red to blue), indicating the largest contrast between the scores for bases paired and those for bases unpaired. This is confirmed by Supplementary Figure S7, which compares the score distributions of bases unpaired, paired with double mutations fully covered, and without fully covered (lack of statistics). These three states are well separated in the distribution of Ps scores in CODA, but neither in mfDCA-RNA, nor in EC-RNA. This confirms the improvement of the signal-to-noise ratio by CODA, due to the fact that CODA employs both functional and non-functional sequences whereas mfDCA-RNA and EC-RNA rely on functional sequences only. If we remove non-functional sequences (defined as relative activity less than one half of the wild type sequence), the performance of CODA will drop drastically with AUC-PR decreasing from 0.53 to 0.39 and MCC from 0.62 to 0.48, indicating that accounting for non-functional sequences is an integral part of the CODA algorithm.

However, the helix regions derived from deep mutation data are much shorter than the native ones (Figure 2A). Undetected base pairs usually occur at the edge of the region with contiguous base pairs because the mutation at the edge of a stem affects less on structural stability and thus leads a weaker covariation signal. Moreover, the mutant library is small and biases toward AT pairs because error-prone PCR tends to have fewer mutations in the GC than in the AT region (47,53). The low mutation coverage leads to low base-pair coverage (or low sensitivity of mfDCA-RNA, EC-RNA and CODA).

To remedy the problem of underestimating the length of contiguous base-paired regions, we introduced a simple base-pair predictor by using experimentally measured base-pair and stacking energies (48) and a restraint that one base cannot be paired with more than a single base. Using energy optimization for determining base-pairing structures also removes the need to set a threshold to separate paired from non-paired bases because such a threshold is not known *a priori*. Only a single adjustable parameter was employed to penalize lone pair and non-AU, GC or wobble GU pairs by setting it to 3 kcal/mol so that the predictor does not produce any lone and base pairs beyond AU, GC and GU in base-pair prediction by Monte Carlo (MC) simulated annealing alone (MC results are the same for CPEB3 to the value between 2 and 5 kcal/mol). As shown in Table 1, MC alone can yield a reasonable performance (although not as accurate as RNAfold in term of the MCC value). Then, we incorporated the pairing score (*Ps*) from CODA as an additional energetic term with a relative weighting factor over the mean Ps scores. Here, we incorporate Ps scores for all possible pairs of bases without using any threshold to pre-define base pairs or exclude any specific pairs. As the Supplementary Figure S8 shows, the performance of CODA+MC is stable after the weighting factor ≥2. We performed MC simulated annealing with random initial seeds 100 times to obtain contact probabilities. Table 1 shows that incorporating CODA to MC increases the number of correctly predicted base pairs from 10 to 25 bp as shown in Figure 2A. A nearly perfect precision-recall curve by CODA+MC (Figure 2B) highlights the power of combining a secondary-structure folding algorithm with CODA analysis. By comparison, we also combined MC with R-scape, mfDCA-RNA and EC-RNA (Table 1) with the optimized weighting factor of 0.8, but 0.5 for R-scape (Supplementary Figure S8). Similar to CODA+MC, R-scape+MC, mfDCA-RNA+MC and EC-RNA+MC yield significant improvement over R-scape, mfDCA-RNA and EC-RNA with MCC = 0.92, 0.90 and 0.93, respectively, although they contain 2–3 false positive predictions (Supplementary Figures S6A and S9A). CODA+MC continues to improve over these two methods with MCC = 0.96.

It is of interest to note that Rfam (56) contains 110 homologous sequences for CPEB3, applying R-scape, mfDCA-RNA and EC-RNA to Rfam sequences yield AUC-PR at 0.09, 0.14 and 0.15 (Supplementary Table S8), respectively. These values are much smaller than to the use of sequences from deep mutations by the same methods (0.30, 0.48 and 0.44, respectively). The result supports the usefulness of randomly mutated sequences to reveal evolutionary coupling.

### Twister ribozyme

**Figure 3.**
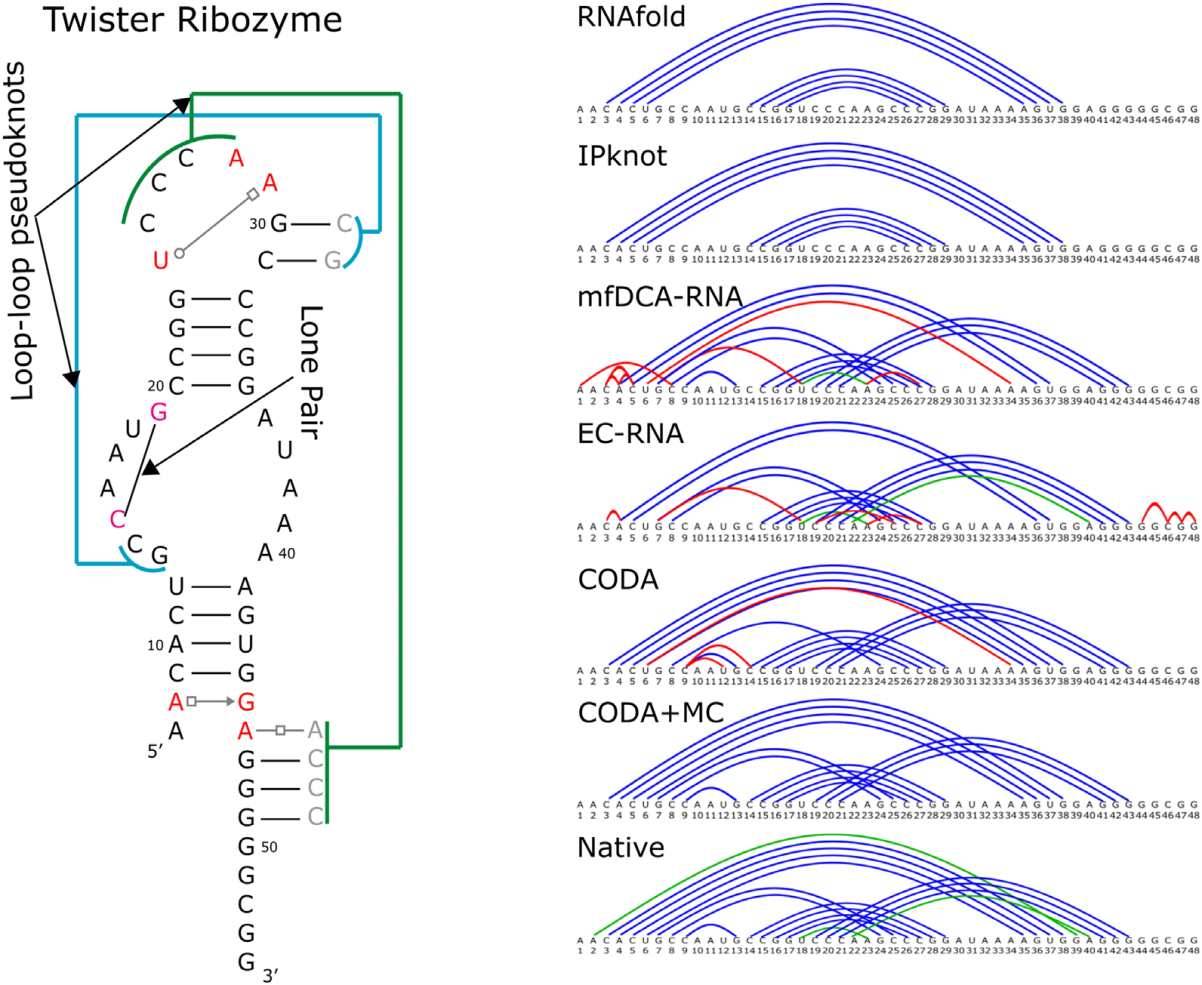
The base-pairing structure of the twister ribozyme along with base pairs predicted by secondary structure predictors RNAfold and IPknot and inferred from its deep mutation data by mean-field direct coupling analysis (mfDCA-RNA), evolutionary couplings (EC-RNA), covariation-induced deviation of activity (CODA), and coupling of CODA and Monte Carlo simulated annealing (CODA+MC). In the right panel, native Watson–Crick and noncanonical base pairs are shown in blue and green, respectively. False positive predictions are shown in red.

To confirm the improvement of CODA analysis over R-scape, EC-RNA and mfDCA-RNA and high-accuracy inference of base-pairing structure by using MC simulated annealing, we obtained the raw deep mutation data of a twister ribozyme from Kobori and Yokobayashi (29). The ribozyme can self-cleave into two RNA fragments of 6 and 48 bases long. The ribozyme mutant library is in the C-terminal 48-nucleotide fragment only. Thus, we will focus on this fragment whose base-pairing structure (57) is shown in Figure 3 (PDB ID: 4OJI). The base pair pattern contains three noncanonical pairs (A2G39, A22A40, and Hoogsteen base pair U18A23), one lone pair (C9G13) as well as two long-distance loop-loop pseudoknots (or kissing interactions) that were considered as tertiary interactions (6,57). The high throughput sequencing data from Kobori and Yokobayashi (29) contains a library of mutants including 144 single, 10 152 double, 240 967 triple and 272 690 other (>3) mutated variants. This is 100% coverage of all possible single and double mutations.

Table 1 compares the performance of various methods for the twister ribozyme. The overall trend is the same as CPEB3 ribozyme. CODA increases 30%, 6% and 19% in AUC-PR over R-scape, mfDCA-RNA and EC-RNA, respectively, and 23%, 20%, 20% in MCC values over R-scape, mfDCA-RNA or EC-RNA. In particular, CODA has 5% and 18% absolute increase in sensitivity and precision, respectively, over either mfDCA-RNA or EC-RNA. For R-scape, CODA has 29% absolute increase in sensitivity but 8% absolute decrease in precision. It is of interest to note that commonly used secondary structure predictors RNAfold and IPknot perform poorly if applying directly to the Twister cleavage fragment (48 bases) with MCC = 0.22 and 0, respectively. RNAfold and IPknot yield comparable performance to R-scape, mfDCA-RNA or EC-RNA only if the full twister sequence is provided for structural folding.

The base-pairing plot (Figure 3) shows that CODA yields overall correct topology with lone pair and pseudoknots but misses 4 bp with three false positive predictions. These false positives are associated with an obvious error of one base paired with more than a single base. Supplementary Figure S10 further compares the precision-recall curve given by R-scape, EC-RNA, mfDCA-RNA and CODA. CODA has zero false positives (100% precision) for the first 7 base pairs (41% sensitivity), indicating that these seven base pairs can be identified without any ambiguity, compared to five by R-scape, six by EC-RNA and five by mfDCA-RNA. The density plot for the base-pairing map (Supplementary Figure S11) and score distributions (Supplementary Figure S12) confirm that the greater separation of bases unpaired and paired is the reason for better performance of CODA, relative to R-scape, EC-RNA and mfDCA-RNA.

More importantly, CODA+MC determines all 14 canonical base pairs at 100% precision, including lone and non-nested base pairs. This further confirms the complementary power of mutation-derived and experimental base-pairing energetic scores. Similar to CPEB3, the combination of MC with R-scape, EC-RNA and mfDCA-RNA (Table 1, Supplementary Figures S6B and S9B) yields significant improvement over R-scape, EC-RNA and mfDCA-RNA, respectively, with a performance much closer to CPEB3 although a different weighting factor of 1 has to be used to maximize the performance of mfDCA-RNA (Supplementary Figure S8). However, unlike CPEB3 ribozyme, CODA+MC missed three noncanonical base pairs (A2G39, A22A40 and Hoogsteen base pair U18A23) whereas EC-RNA+MC and mfDCA-RNA+MC captured A22A40 with one false positive in the same stem. It is noted that Rfam contains (56) only 31 homologous sequences for twister, too few to achieve a reasonable performance by R-scape, EC-RNA and mfDCA-RNA.

One interesting question is how the performance of CODA depends on the total number of reads. Supplementary Figure S13 shows that the MCC value as a function of the total number of reads by randomly removing the reads in the twister data. All of R-scape, mfDCA-RNA and EC-RNA show large fluctuation whereas the MCC value of CODA is stable ∼0.75 from 5% (10^6.5^) to 100% (10^7.8^) reads. This result suggests that CODA is more robust against the size variation of the mutation data.

For CODA analysis, we have included triple mutants for those position pairs not covered by double mutations. As shown in Supplementary Table S9, there is a significant reduction in the performance in CPEB3 if only single and double mutants are utilized. AUC-PR reduced from 0.53 to 0.42 and MCC from 0.62 to 0.52. This indicates the utility of triple mutants when the coverage of double mutations is low, similar to the beneficial use of all mutants in R-scape, EC-RNA and mfDCA-RNA.

## DISCUSSION

Base pairing is responsible for stabilizing overall structural fold of RNA structures and the key for understanding functional mechanisms. A full base-pairing structure is more than secondary structure because it contains tertiary contacts such as lone base pairs and kissing loop pseudoknots. This work shows that these challenging tertiary contacts can be inferred from deep mutation data by analyzing covariation-induced deviation of double-mutation activity from the independent-mutation model (CODA). The combination of CODA with a Monte Carlo simulated annealing leads to detection of all WC pairs (except non-WC pairs but including lone WC pairs) for twister ribozyme at 100% precision and all WC pairs plus two non-WC pairs for CPEB3 ribozyme at 96% precision, despite that CPEB3 ribozyme has only a small library of <50 000 single-to-triple mutants and the mutation data of twister ribozyme is limited to the large fragment from self-cleavage. Moreover, two pseudoknots associated with tertiary interactions in CPEB3 and twister were both captured.

The CODA analysis developed here has a better performance than R-scape, EC-RNA and mfDCA-RNA. This is reflected from 19% and 5% increases in MCC by CODA over the next best (mfDCA-RNA) for CPEB3 and twister, respectively. More importantly, CODA is more robust against changes in the number of reads (Supplementary Figure S13) and the weighting factor when combining with MC simulated annealing (Supplementary Figure S8). This improvement in performance is likely due to an increase in the signal-to-noise ratio as a result of employing all mutation variants functional or non-functional in CODA as shown in Supplementary Figures S7 and S12. It should be mentioned that this comparison is not entirely justifiable because EC-RNA and mfDCA-RNA were developed for diverging mutations rather than close homologs of a few mutations. A more justifiable comparison is made to epistasis analysis below.

Identification of noncanonical, lone and non-nested base pairs is not a trivial exercise. Double-stranded long helical stems with many stacked base pairs are relatively easy to detect because they serve as core structural elements and a mutation at the center of these stems will have a large impact on the overall structural stability. Pseudoknots, on the other hand, are often involved with a few base pairs between two distant hairpin loops (loop-loop pseudoknots or kissing stem-loop (6,58)) as shown in Figures 1 and 3. These base pairs are considered as a part of tertiary contacts to help to stabilize the overall 3D shape. CODA as well as evolutionary or direct coupling analysis can capture one or more base pairs within a region of contiguous base pairs regardless if the pair is nested or non-nested (pseudoknots). In other words, it offers an excellent base-pairing topology for expanding into the full base-pairing structure. This is confirmed by a combination of CODA or evolutionary/direct coupling with MC simulated annealing, which significantly increases sensitivity while maintaining high precision.

A total of three base pairs were missed by the combination of CODA and Monte Carlo simulated annealing in twister ribozyme, all of which are noncanonical pairs. They are A2G39, A22A40 and Hoogsteen base pair U18A23. The backbone orientations of these noncanonical pairs are quite different from Watson–Crick pairs so that all double mutations on these sites yield negative signals in CODA. Moreover, these three noncanonical base pairs are all at the edge of a helical stem (Figure 3). Their mutations may not necessarily have a detrimental effect on the ribozyme's function. By comparison, all three noncanonical base pairs in CPEB3 ribozyme (G6U41, C12A35 and U26U43) have positive signals in CODA although only two have signals strong enough to be detectable by the combination of CODA and MC simulated annealing. One of the noncanonical base pairs is a lone pair in pseudoknot, which may have played an important role in the overall stability of the ribozyme's structure. Interestingly, mfDCA-RNA+MC and EC-RNA+MC can capture one noncanonical base pair with one false positive in twister as well as one or two noncanonical base pairs in CPEB3 with some false positives (Supplementary Figure S9), suggesting that it is possible to further enhance the signals of noncanonical base pairs in CODA. Because canonical and noncanonical base pairs are treated equally in CODA, a CODA version that treats canonical and noncanonical base pairs separately will likely improve the sensitivity of detecting noncanonical base pairs. However, this separation will increase the risk of over training and is a good subject for a future study when more data is available.

This study investigated two self-cleaving ribozymes as proof of concept. There is a question if the parameters used in CODA+MC could have overfitted the two ribozymes tested here. The independent-mutation model in CODA is an unsupervised model generated from non-linear regression between single and double mutations with the parameters optimized to produce the best fit between predicted and actual relative activities of double mutants. We have employed the default *C* and γ parameters (*C* = 2000 and γ = 2.0) where *C* is a hyperparameter to control how much to penalize the misclassification and γ is a hyperparameter for the non-linear Gaussian kernel. In addition, we define outliers (base pairs) according to 3 standard deviations (SD) away from the fitted model. Supplementary Table S7 shows the variation of maximal MCC for CPEB3 as we change the number of SDs from 1 to 7, *C* from 1 to 10 000, and γ from 0.01 to 10 while fixing the other two values at their default values. MCC values change little in a wide range tested. This result confirms the robustness of the model obtained.

For MC simulated annealing, one parameter introduced is the energy penalty that discourages lone pairs and noncanonical pairs by MC alone. The parameters for simulated annealing were chosen to ensure the identification of the global minimum within a reasonable computing time. A value between 2–5 kcal/mol for the energy penalty makes no changes to the outcome of MC alone. Another parameter is the weighting parameter for the CODA score (or R-scape, EC-RNA and mfDCA-RNA scores) before mixing with experimentally measured base-pairing energy scores. Supplementary Figure S8 confirms that the CODA+MC method is more robust than R-scape+MC, EC-RNA+MC and mfDCA+MC as the latter two methods achieve their best performance within a narrow range of the weighting factor (∼0.8–1) while CPEB3 has a stable performance for the weighting factor ≥2. The robustness of CODA is also reflected from the fact that its performance is independent of the total number of reads over a wider range than EC-RNA and mfDCA-RNA (Supplementary Figure S13). However, to truly prove the general applicability of CODA to other RNAs, more experimental studies for RNAs with known structures or RNAs with unknown structures have to be performed and validated. Participation of RNA puzzles (blind prediction) (25) will be the ultimate test for future consideration. Nevertheless, this work clearly demonstrates the potential in utilizing deep mutation data for pinpointing both secondary and tertiary base-pair contacts as multiple methods (R-scape+MC, EC-RNA+MC, mfDCA-RNA+MC and CODA+MC) can achieve highly accurate performance (MCC ≥ 0.9).

Obtaining structural clues of self-cleaving ribozymes are important because self-cleaving ribozymes are broadly distributed in genomes of different organisms from viroids to vertebrates (40–42). Understanding their structures and functions is only at the beginning (37) with almost all the known ribozymes having interesting mechanistic differences (37–39). This deep-mutation-based method can infer base-pairing structures at the single base-pair level with sensitivity and precision inaccessible to current multi-dimensional chemical probing methods.

The method, however, is not limited to self-cleaving ribozymes. Previously mutational scanning was applied to investigate tRNA (28), RNA catalysis (26,59), RNA-protein interactions (31,32), and RNA-RNA interactions (27,30). The high-throughput techniques employed for function selection prior to sequencing includes *in vitro* affinity/activity selection (26,31–33) and *in vivo* assay according to cell growth (28,30) and fluorescence intensity (60). The success of these ingenious *in vitro* and *in vivo* techniques for function selections indicates the wide applicability of deep mutational scanning in determining *in vitro* and *in vivo* base-pairing structures of many types of RNAs beyond self-cleaving ribozymes. Not all RNAs will be suitable for high-throughput deep mutation analysis, however. The method will require an RNA whose phenotype is strongly depending on its unique structure. This phenotype has to be measured quantitatively by a high-throughput technique. The effect of RNA modification on function is not considered. In addition, not all noncanonical base pairs were detected for twister and CPEB3. Furthermore, base triplets, although rare (61), are simply ignored in secondary structure folding. Nevertheless, a deep mutational analysis will make more RNAs accessible for high-quality determination of base pairs. The technique will be complementary to, but certainly not as a mean for the replacement of existing structure-determination technologies.

Another limitation for deep mutation studies is RNA sequence length. A long RNA would have to overcome an increased difficulty in amplification of RNA libraries with high mutation rates, reduced coverage due to fast increase in the number of possible double mutations, decreased quality of reads for longer RNAs, and the reduced ability to reach the global minimum for long RNAs by MC simulated annealing. We demonstrated that the CODA analysis is capable of detecting nested and non-nested base pairs as well as some noncanonical base pairs even from a small mutant library of <5 × 10^4^ mutants for 81-nucleotide CPEB3 ribozyme. This suggests that the method may be applicable to RNAs with 800 bases or less for an achievable library of 5 × 10^6^ mutants (or 600 bases due to the current read length limit of the next-generation sequencing platforms). While this number may be a theoretical upper bound, RNAs of 200–300 nucleotides are certainly approachable.

However, it is important to note that combining CODA with MC simulated annealing can fold the complex base-pairing pattern of the twister *fragment* correctly. By comparison, the secondary structure predictors RNAfold and IPknot have to use the whole sequence to achieve a reasonable prediction. For example, MCC = 0.22 if using the twister fragment sequence for RNAfold, compared to 0.68 when using the whole twister sequence (Table 1). Solving fragment base-pairing structures may be an approach for probing those long RNAs if proved challenging to perform deep mutation on the whole chain.

In addition to its own importance, the accurate, full base-pairing structure obtained from deep mutational scanning should benefit high-quality 3D structure modeling. This is because a full base-pairing structure contains not only secondary structure motif but also important tertiary contacts of lone, noncanonical, and non-nested base pairs. Thus, it can serve as a preformed, quasi-three-dimensional frame for correctly folding into the right RNA tertiary structure. Its usefulness for 3D structure modeling has been demonstrated in RNA Puzzles (blind RNA structure prediction) even with the data not yet at the single-base-pair level (25). We have attempted 3D structure prediction by using Rosetta (62) and 3dRNA (63) and found that the number of restraints is not large enough to fold the sophisticated topology of twister. We will defer this to future studies.

Moreover, the CODA analysis should be a powerful tool for analyzing deep mutation data of proteins. This transferability is reflected from migration of protein mutational coupling analysis programs (EC and mfDCA) to RNAs although they were originally designed for proteins. Hydrogen-bonded base-pairing interactions in RNA structures are the dominant interaction with the strongest covariation signals. As a result, what emerges from the CODA analysis will be the base-base contact map in term of base pairing. On the other hand, hydrophobic packing with complementary shapes and sizes of side chains is the dominant driving force for protein folding (64). Thus, CODA analysis will lead to residue-residue contact maps based on distance proximity of amino acid residues. Because an accurate determination of protein contact maps has been demonstrated to yield high-resolution protein structure (65), the results reported here suggest the potential of protein structural inference from deep mutational scanning (66,67).

Indeed, after the completion of this work, Rollins *et al.* (50) and Schmiedel and Lehner (49) published their studies of employing deep mutational scanning for highly accurate inference of several protein contact maps for protein folding. In particular, Rollins *et al.* (50) further applied their epistasis method to twister ribozyme. For completeness, we obtained the result of Rollins *et al.* (Epi-Rollins for short) and implemented the epistasis program of Schmiedel and Lehner (Epi-SL for short) with minor adaption for RNA. Their results for the Twister fragment, shown in Supplementary Table S10 with corresponding base-pairing structures shown in Supplementary Figure S14. AUC-PR and MCC are 0.50 and 0.59, respectively, by Epi-Rollins, 0.59 and 0.67, respectively, by Epi-SL, compared to 0.72 and 0.79, respectively, by CODA. The combination of Epi-SL with MC simulated annealing with a specifically optimized a weight of 0.8 improves over Epi-SL with MCC = 0.91, matching the performance by CODA+MC. The matching performance, however, appears only at weight of 0.8 whereas the performance of CODA+MC is stable for weight ≥2 (Supplementary Figure S8). Nevertheless, the result further confirms that highly accurate structural inference can be achieved by coupling statistical covariation analysis of deep mutation data with a secondary-structure folding algorithm.

## DATA AVAILABILITY

Illumina sequencing data for the CPEB3 ribozyme were submitted to the NCBI Sequence Read Archive (SRA) under SRA accession number PRJNA515794 (https://www.ncbi.nlm.nih.gov/sra/PRJNA515794).

All custom scripts needed to repeat the analyses are available at https://github.com/zh3zh/CODA. The code takes about 1 hour for preprocessing sequencing data, 1 second for generating CODA values and pairing probabilities, and another hour for Monte Carlo simulated annealing using a workstation with 16 core (2.6GHZ) and a maximum RAM of 128G.

## Supplementary Material

gkz1192_Supplemental_File
