# Supplementary material for: Accurate inference of the full base-pairing structure of RNA by deep mutational scanning and covariation-induced deviation of activity
Source: Nucleic Acids Res. 2019 Dec 24;48(3):1451–65. doi: 10.1093/nar/gkz1192 (PMC7026644; doi:10.1093/nar/gkz1192)
Supplement: gkz1192_Supplemental_File [file gkz1192_supplemental_file.docx]

# Supplementary Materials for

# Accurate inference of the full base-pairing structure of RNA by deep mutational scanning and covariation-induced deviation of activity

## Table S1. Oligonucleotides used in this study.

| Name | Sequence (5’ - 3’) | Notes |
| --- | --- | --- |
| CPEB3_wt | TAATACGACTCACTATAGGGATAACAGGGGGCCACAGCAGAAGCGTTCACGTCGCAGCCCCTGTCAGATTCTGGTGAATCTGCGAATTCTGCTGTATATCTCATTTG  AGTTACTCAGGATTACTGGCCGTCGTTTTAC | DNA template. |
| CPEB3_C57U | TAATACGACTCACTATAGGGATAACAGGGGGCCACAGCAGAAGCGTTCACGTCGCAGCCCCTGTCAGATTCTGGTGAATCTGTGAATTCTGCTGTATATCTCATTTG  AGTTACTCAGGATTACTGGCCGTCGTTTTAC | DNA template. |
| M13F | GTAAAACGACGGCCAGT | PCR primer. |
| T7prom | TAATACGACTCACTATAGGG | PCR primer. |
| RT_m13f_adp1 | CCCTACACGACGCTCTTCCGATCTGTAAAACGACGGCCAGT | Reverse transcription primer. |
| TSO | CTCGGCATTCCTGCTGAACCGCTCTTCCGATCTrGrGrG | Template switching oligo. |
| Bar_F | TAATACGACTCACTATAGGGA | PCR primer for adding barcode. |
| Bar_R | GTAAAACGACGGCCAGTNNNNNNNNNNNNNNNAATCCTGAGTAACTCAAAT | PCR primer for adding barcode. |
| P5R1_m13f | AATGATACGGCGACCACCGAGATCTACACTCTTTCCCTACACGACGCTCTTCCGATCTGTAAAACGACGGCCAGT | PCR primer for DNA-seq library preparation. |
| P7R2_t7p | CAAGCAGAAGACGGCATACGAGATCGGTCTCGGCATTCCTGCTGAACCGCTCTTCCGATCTTAATACGACTCACTATAGG | PCR primer for DNA-seq library preparation. |
| P5R1_adp1 | AATGATACGGCGACCACCGAGATCTACACTCTTTCCCTACACGACGCTCTTC | PCR primer for RNA-seq library preparation. |
| P7R2_adp2 | CAAGCAGAAGACGGCATACGAGATCGGTCTCGGCATTCCTGCTGAAC | PCR primer for RNA-seq library preparation. |
| CP_S | /56-FAM/rArUrArArCrArGrGrGrGrGrCrCrArC/36-TAMSp/ | Modified RNA oligo for fluorescence-based activity assay. |
| CP_E_wt_for | TAATACGACTCACTATAGAGCAGAAGCGTTCACGTCGCAGCCCCTGTCAGATTC | PCR primer for fluorescence-based activity assay. |
| CP_E_U26A_for | TAATACGACTCACTATAGAGCAGAAGCGTACACGTCGCAGCCCCTGTCAGATTC | PCR primer for fluorescence-based activity assay. |
| CP_E_U26C_for | TAATACGACTCACTATAGAGCAGAAGCGTCCACGTCGCAGCCCCTGTCAGATTC | PCR primer for fluorescence-based activity assay. |
| CP_E_A45G_for | TAATACGACTCACTATAGAGCAGAAGCGTTCACGTCGCAGCCCCTGTCGGATTC | PCR primer for fluorescence-based activity assay. |
| CP_E_A45U_for | TAATACGACTCACTATAGAGCAGAAGCGTTCACGTCGCAGCCCCTGTCTGATTC | PCR primer for fluorescence-based activity assay. |
| CP_E_A18G_for | TAATACGACTCACTATAGAGCGGAAGCGTTCACGTCGCAGCCCCTGTCAGATTC | PCR primer for fluorescence-based activity assay. |
| CP_E_wt_rev | CTCTTCGCTCTTCCAGCAGAATTCGCAGATTCACCAGAATCTGACAGGGGCTGC | PCR primer for fluorescence-based activity assay. |
| CP_E_A45G.U60A_rev | CTCTTCGCTCTTCCAGCAGAATTCGCTGATTCACCAGAATCCGACAGGGGCTGC | PCR primer for fluorescence-based activity assay. |
| CP_E_A45G.U60C_rev | CTCTTCGCTCTTCCAGCAGAATTCGCGGATTCACCAGAATCCGACAGGGGCTGC | PCR primer for fluorescence-based activity assay. |
| CP_E_A45G_rev | CTCTTCGCTCTTCCAGCAGAATTCGCAGATTCACCAGAATCCGACAGGGGCTGC | PCR primer for fluorescence-based activity assay. |
| CP_E_A45U.U60A_rev | CTCTTCGCTCTTCCAGCAGAATTCGCTGATTCACCAGAATCAGACAGGGGCTGC | PCR primer for fluorescence-based activity assay. |
| CP_E_A45U.U60C_rev | CTCTTCGCTCTTCCAGCAGAATTCGCGGATTCACCAGAATCAGACAGGGGCTGC | PCR primer for fluorescence-based activity assay. |
| CP_E_A45U_rev | CTCTTCGCTCTTCCAGCAGAATTCGCAGATTCACCAGAATCAGACAGGGGCTGC | PCR primer for fluorescence-based activity assay. |
| CP_E_U69C_rev | CTCTTCGCTCTTCCAGCGGAATTCGCAGATTCACCAGAATCTGACAGGGGCTGC | PCR primer for fluorescence-based activity assay. |
| CP_E_U69G_rev | CTCTTCGCTCTTCCAGCCGAATTCGCAGATTCACCAGAATCTGACAGGGGCTGC | PCR primer for fluorescence-based activity assay. |

## Table S2. Summary of deep sequencing results of CPEB3 ribozyme in three batches of experiments.

|  | Number of DNA-seq reads | Number of Barcodes | Number of RNA-seq reads | Cleaved | Uncleaved |
| --- | --- | --- | --- | --- | --- |
| Batch 1 | 9,834,235 | 54,502 | 16,490,545 | 41.4% | 58.6% |
| Batch 2 | 28,107,628 | 89,397 | 32,061,733 | 17.8% | 82.2% |
| Batch 3 | 23,028,064 | 115,977 | 90,995,324 | 49.4% | 50.6% |

## Table S3. Fractions of CPEB3 ribozyme mutants in three batches of experiments

| Number of Mutations | Batch 1 | Batch 2 | Batch 3 |
| --- | --- | --- | --- |
| 0 | 28.5% | 0.9% | 5.3% |
| 1 | 30.6% | 4.0% | 14.5% |
| 2 | 22.1% | 9.8% | 22.2% |
| 3 | 12.0% | 16.0% | 22.1% |
| 4 | 4.9% | 19.6% | 16.9% |
| 5 | 1.5% | 18.7% | 10.5% |
| 6 | 0.4% | 14.3% | 5.3% |
| 7 | 0.1% | 9.4% | 2.3% |
| 8 | 0.0% | 5.0% | 0.8% |
| 9 | 0.0% | 2.2% | 0.3% |

## Table S4. Mutation rates of CPEB3 ribozyme in three batches of experiments

| Wild type | Batch 1 | Batch 2 | Batch 3 |
| --- | --- | --- | --- |
| A | 2.96% | 8.86% | 6.18% |
| U | 3.39% | 8.84% | 6.35% |
| G | 0.50% | 2.97% | 1.57% |
| C | 0.40% | 2.59% | 1.37% |
| all | 1.73% | 5.62% | 3.7% |

Table S5. The minimum, median, and maximum of reads of detected variants.

|  | B1 | B2 | B3 |
| --- | --- | --- | --- |
| Max | 6589330 | 719014 | 20328858 |
| Min | 5 | 5 | 5 |
| Median | 238 | 392 | 267 |

## Table S6. The number of variants and the coverage of double mutations of CPEB3 ribozyme in three batches of experiments

| # Mutations | 1 | 2 | 3 | 4 | 5 | Double mutation coverage1 | Double mutation coverage2 |
| --- | --- | --- | --- | --- | --- | --- | --- |
| Batch 1 | 239 | 4781 | 4897 | 1771 | 476 | 16.4% | 25.1% |
| Batch 2 | 240 | 5161 | 11534 | 12165 | 9651 | 17.7% | 36.7% |
| Batch 3 | 243 | 9566 | 22409 | 15933 | 8498 | 32.8% | 52.9% |
| Merged | 243 | 11968 | 36214 | 29657 | 18567 | 41.0% | 61.3% |

- Double mutation coverage1: only use information from double mutants
- Double mutation coverage2: use information from both double mutants and triple mutants, for triple mutant XYZ, *RA(XY) = RA(XYZ)/RA(Z)* when *RA(Z)>0.5*.

## Table S7. Performance of CODA measured by MCC as a function of three parameters: C and γ in the non-linear Gaussian kernel of our Support Vector Regression (SVR) model and the definition of outliers (predicted versus measured relative activities) according a given number of standard deviation (#SD) away from the fitted regression model. Only one variable is changed while the other two are fixed at default values. The default value we employed for #SD, C and γ are 3, 2000 and 2.

| #SD | MCC | C | MCC | γ | MCC |
| --- | --- | --- | --- | --- | --- |
| 1 | 0.62 | 1 | 0.62 | 0.01 | 0.62 |
| 2 | 0.62 | 10 | 0.62 | 0.1 | 0.62 |
| 3 | 0.62 | 100 | 0.62 | 0.5 | 0.62 |
| 4 | 0.63 | 1000 | 0.62 | 1 | 0.62 |
| 5 | 0.65 | 2000 | 0.62 | 2 | 0.62 |
| 6 | 0.65 | 3000 | 0.62 | 3 | 0.62 |
| 7 | 0.65 | 4000 | 0.62 | 4 | 0.62 |
|  |  | 5000 | 0.62 | 5 | 0.62 |
|  |  | 10000 | 0.62 | 10 | 0.62 |

##

## Table S8. Performance of three mutational coupling analysis (R-scape, mfDCA-RNA and EC-RNA) using homologous sequences in 110 Rfam sequences for CPEB3 family.

| Method | AUC_PR | MCC | Sensitivity | Precision |
| --- | --- | --- | --- | --- |
| RFAM-R-scape | 0.09 | 0.29 | 0.31 | 0.28 |
| RFAM-mfDCA-RNA* | 0.14 | 0.36 | 0.31 | 0.42 |
| RFAM-EC-RNA | 0.15 | 0.28 | 0.08 | 1.00 |

## * Average product correction (apc) was used to improve the performance of mfDCA-RNA.

Table S9. Performance of CODA for CPEB3 ribozyme using deep mutation data with or without triple mutations (number of mutations ≤2 or ≤3)

| Method | AUC_PR | MCC | Sensitivity | Precision |
| --- | --- | --- | --- | --- |
| ≤2 | 0.42 | 0.52 | 0.27 | 1.00 |
| ≤3 | 0.53 | 0.62 | 0.38 | 1.00 |

## Table S10. Performance of Epistasis analysis for twister ribozyme given by Rollins et al (Epi-Rollins) and by Schmiedel and Lehner (Epi-SL). Epi-Rollins for twister is directly from Rollins’s deposited data. Epi-SL was implemented in our local machine with minor modification for RNA.

| Method | AUC_PR | MCC | Sensitivity | Precision |
| --- | --- | --- | --- | --- |
| Epi-Rollins^a^ | 0.50 | 0.59 | 0.35 | 1.00 |
| Epi-SL | 0.59 | 0.67 | 0.59 | 0.77 |
| Epi-SL+MC ^b^ | 0.90 | 0.91 | 0.88 | 0.94 |
| CODA+MC | 0.91 | 0.91 | 0.82 | 1.00 |

## ^a^based on the sum of epistasis score. The result based on the maximal value of epistasis score is similar.

## ^b^specifically optimised weight for mixing epistasis scores with experimentally measured base-pairing energy scores as in R-scape+MC, EC-RNA+MC, mfDCA-RNA+MC, and CODA+MC.


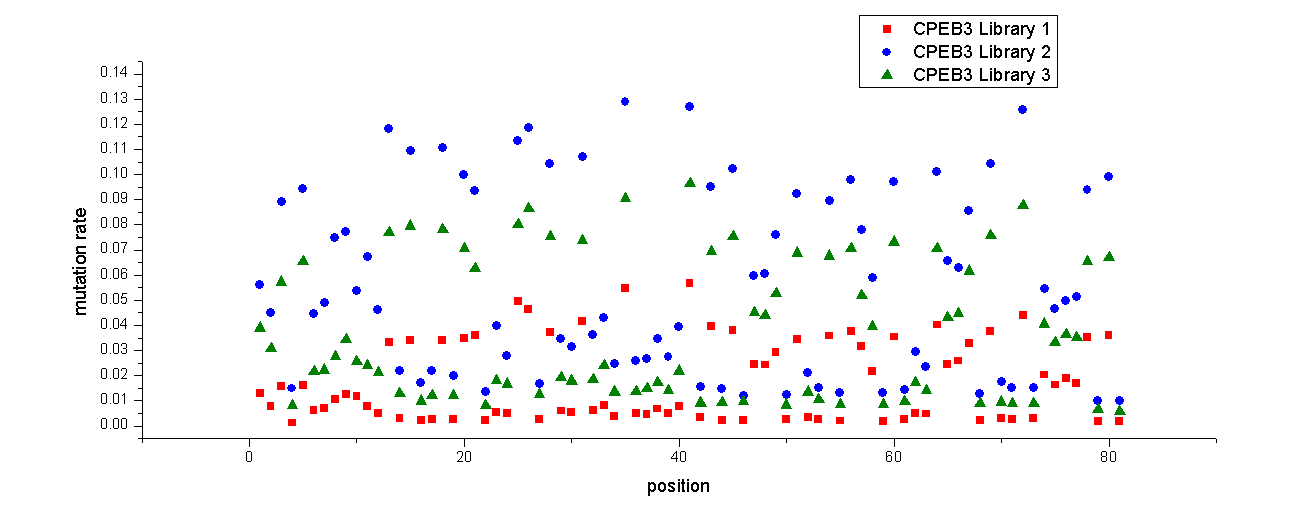


Figure S1. Mutation rates of three batches at each nucleotide position of CPEB3 ribozyme.


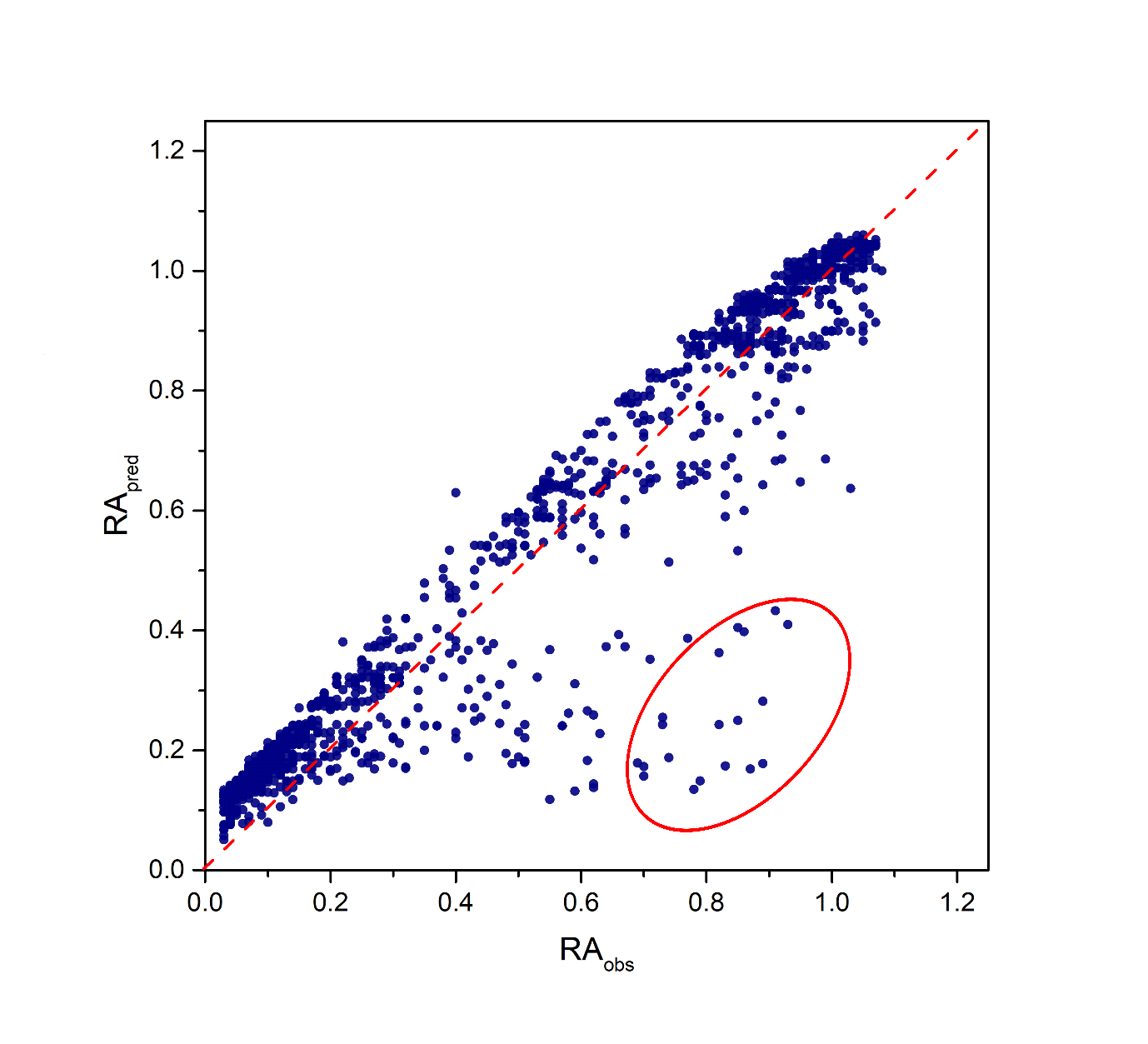


Figure S2. Observed relative activities (*RA*) of the double mutants of twister ribozyme are compared to predicted *RA* by the regression model, the red circle indicates the outliers with significant covariation-induced deviations of activity from the regression model.


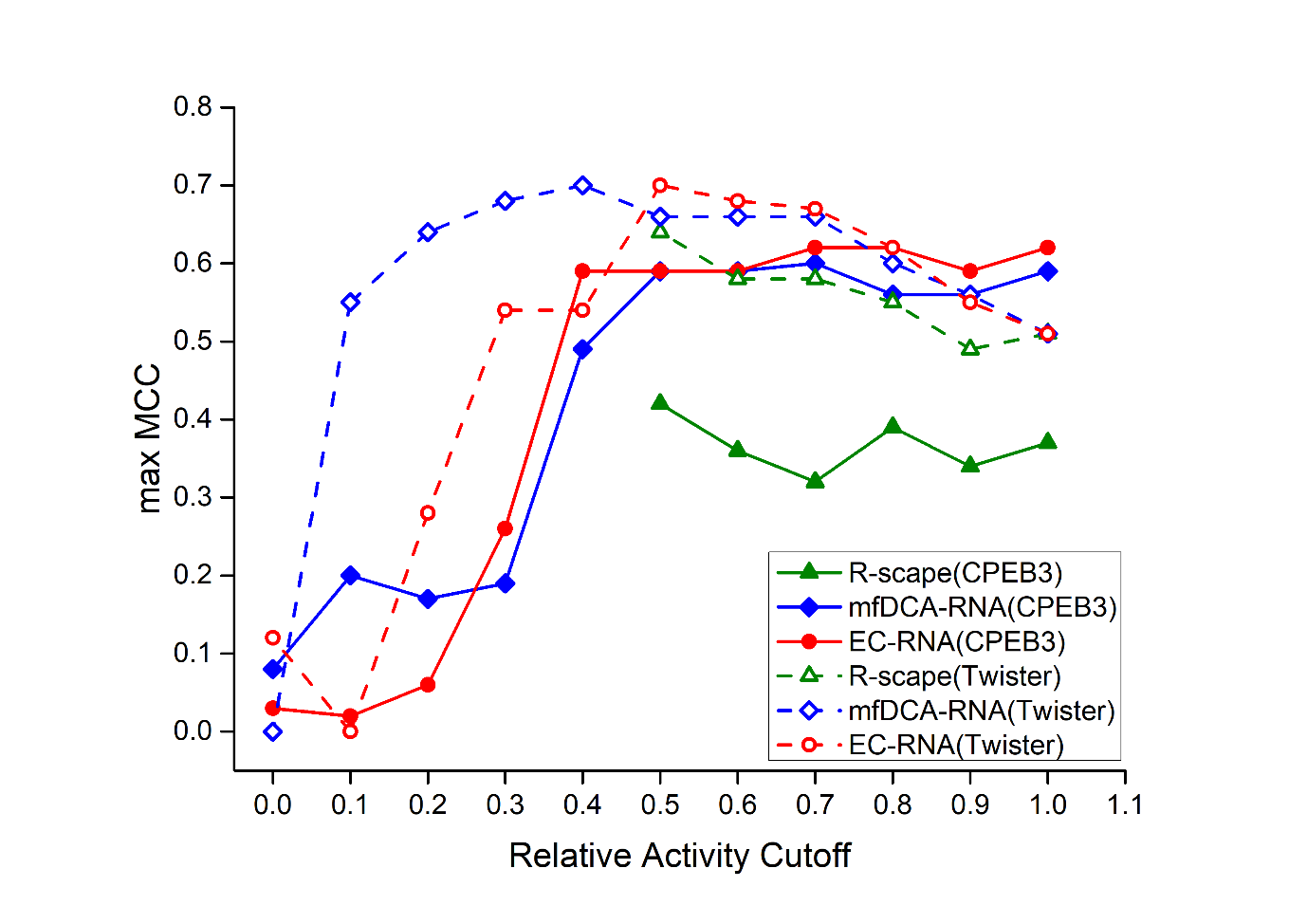


Figure S3. The performance of R-scape, mfDCA-RNA and EC-RNA in term of Matthews correlation coefficient as a function of the cut-off for defining functional ribozymes (twister and CPEB3 ribozymes as labelled). We failed to get results of R-scape when using low relative activity cut-off (< 0.5).


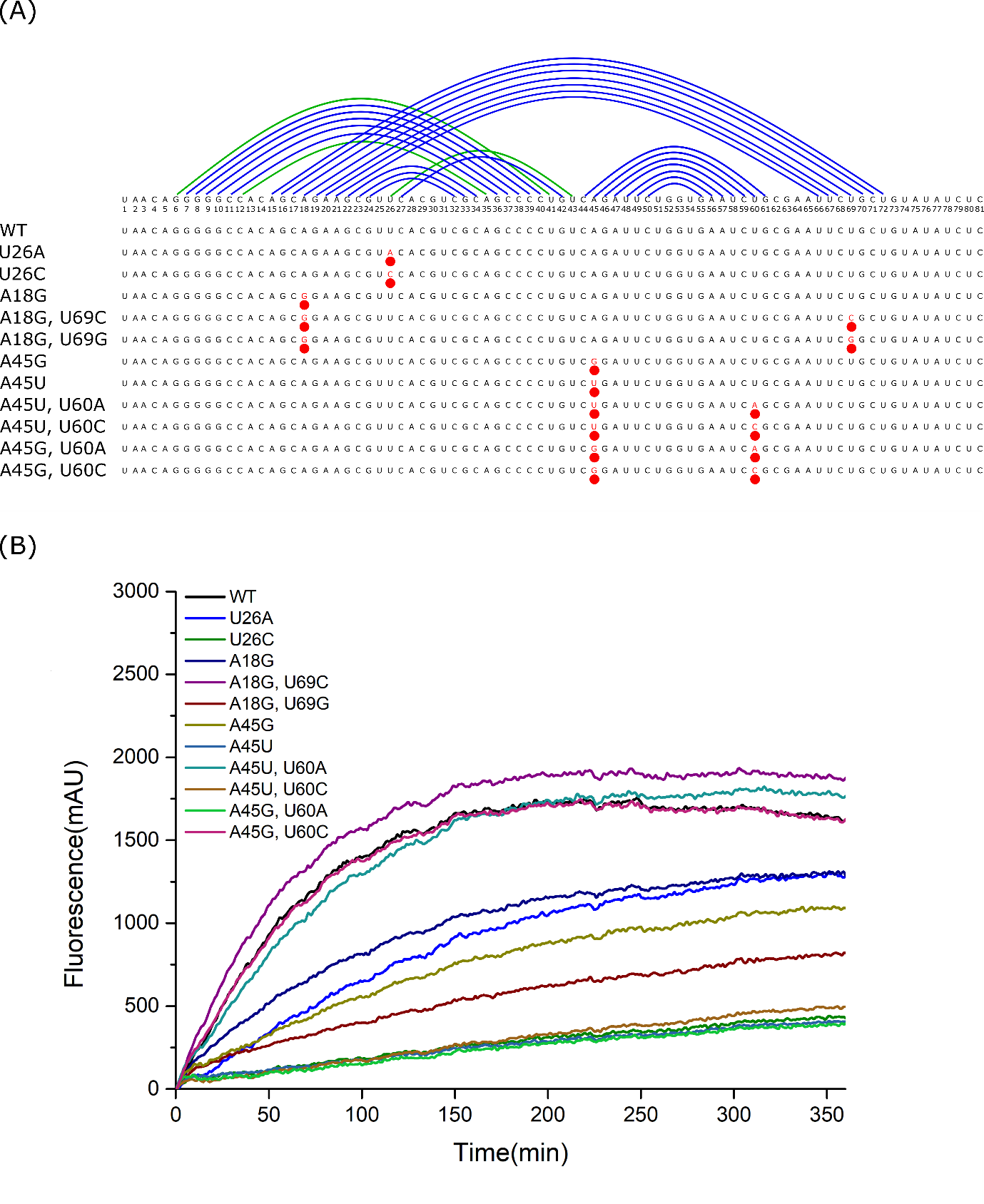


Figure S4. Kinetic analysis of the wild type CPEB3 ribozyme and the 11 selected mutants. (A) Schematic diagram of 12 sequences. The mutations to wild-type sequence are shown in red color and labelled with red dots. (B) Cleavage kinetics of the 12 sequences. The kinetics was measured by the fluorescence assay.


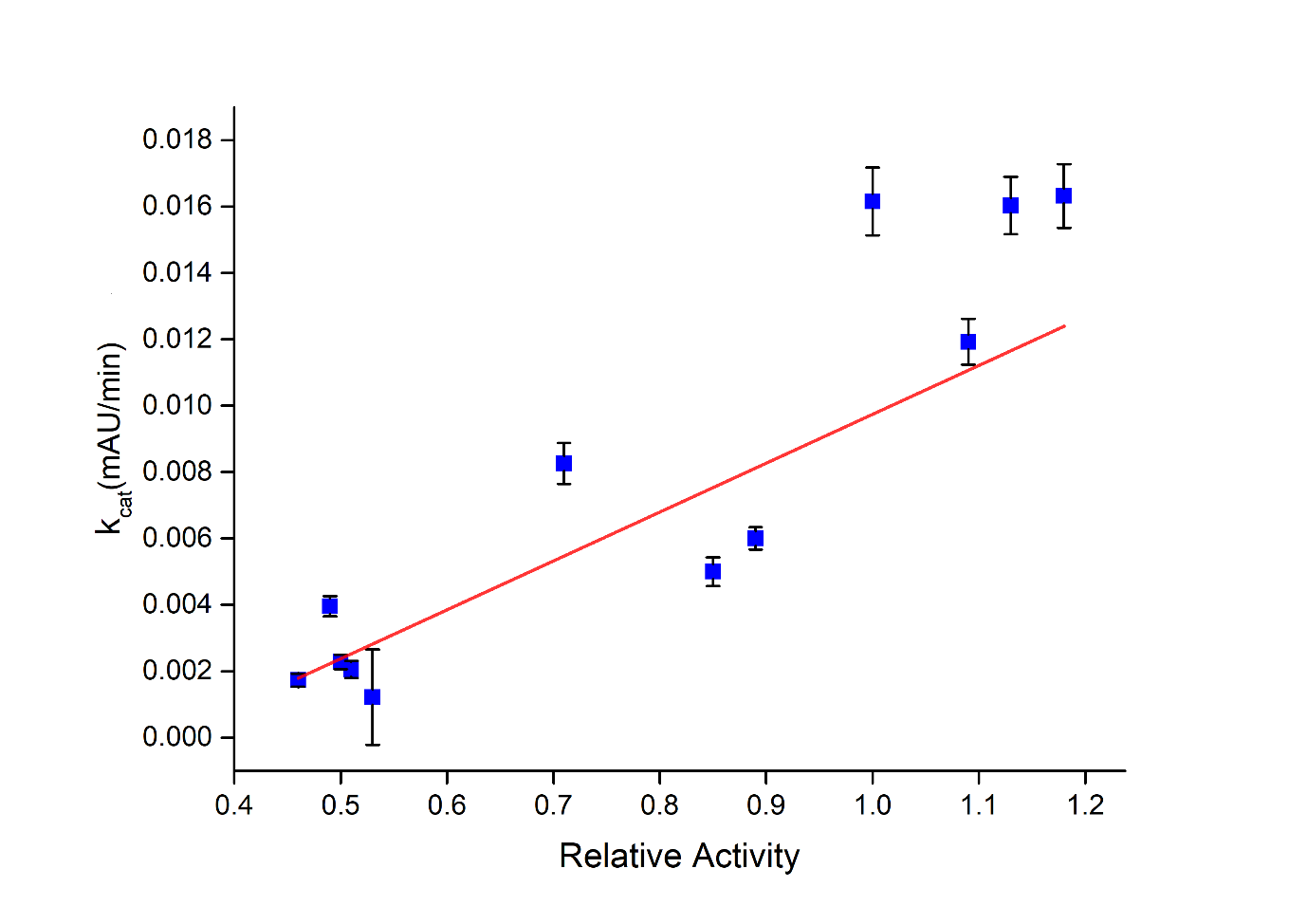


Figure S5. The relative activity from deep sequencing data (x-axis) versus *k*_cat_ from kinetic analysis (y-axis). Each dot represents one mutant. The data point is the average of *k*_cat_ from four independent samples, with error bars denoting their standard deviation. The Pearson correlations between the relative activity and the mean of *k*_cat_ is 0.914.


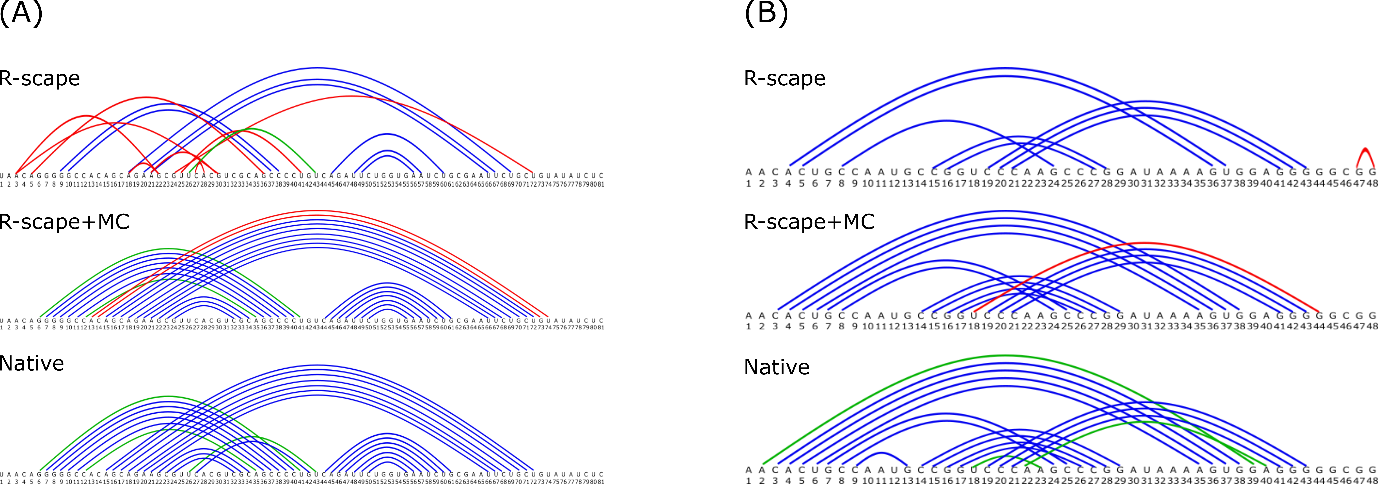


Figure S6. The base-pairing structure of the CPEB3 and twister ribozyme predicted by R-scape with MC simulated annealing (weighting factor of 0.5). (A) CPEB3 (B) twister ribozyme. Native Watson-Crick and noncanonical base pairs are shown in blue and green, respectively. False positive predictions are shown in red.


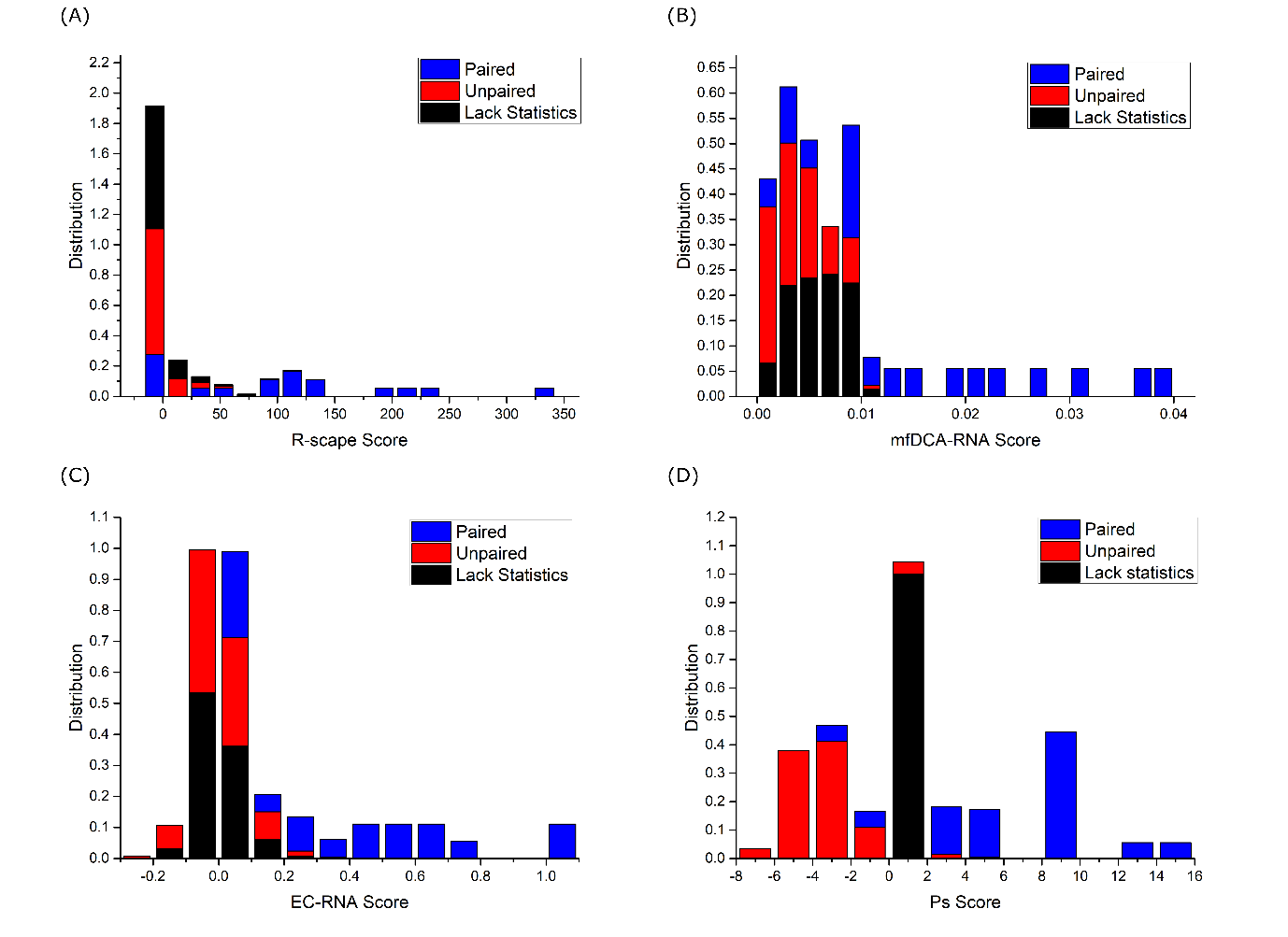


Figure S7. Distributions of R-scape (A), mfDCA-RNA (B), EC-RNA (C) and Pairing Ps (CODA) (D) scores for bases paired, unpaired, and lack of statistics in CPEB3 ribozyme, respectively.


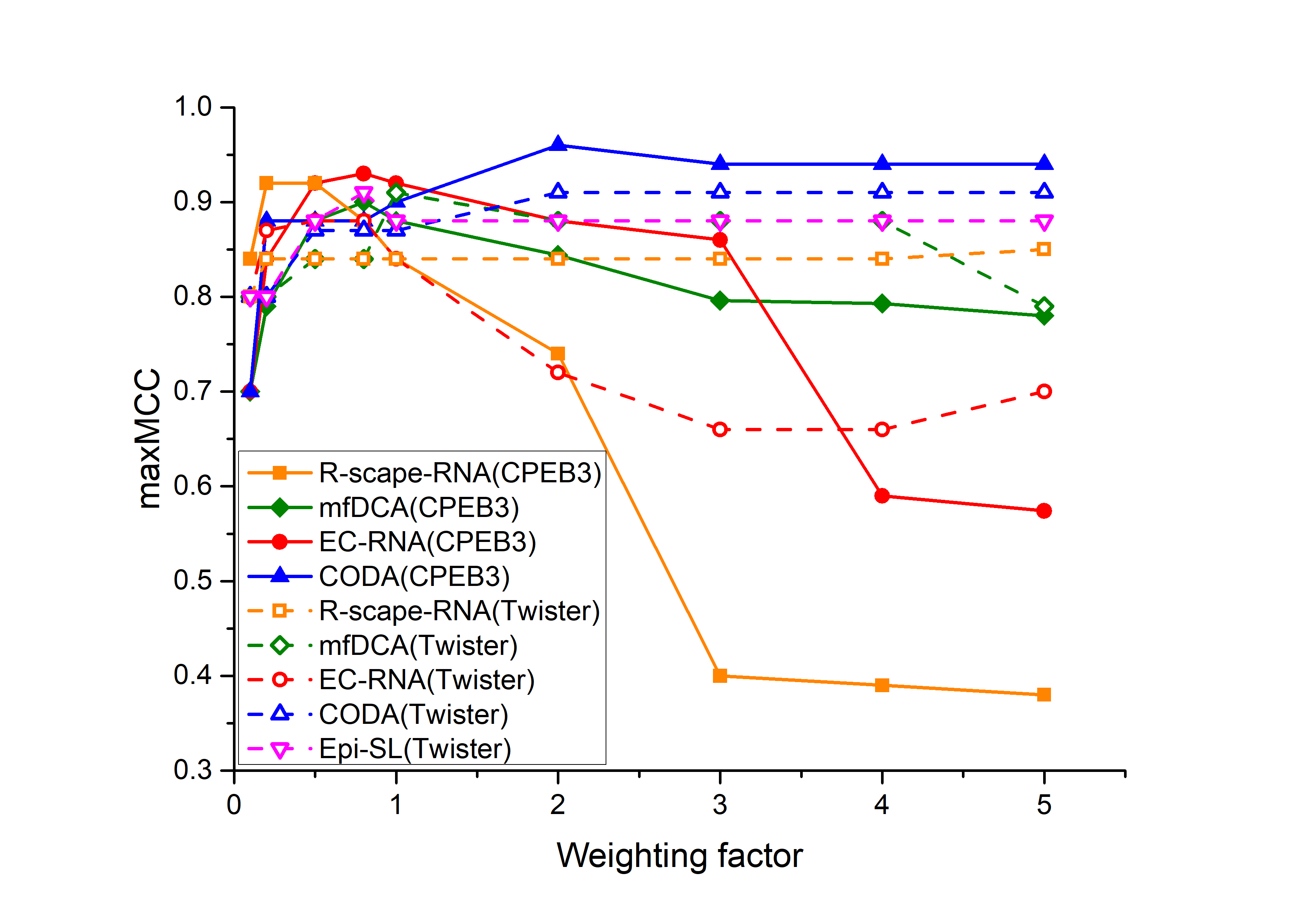


Figure S8. The effect of the weighting factor on mutation-derived scores on the performance of Monte-Carlo simulated annealing in term of Matthews correlation coefficient on CPEB3 and twister ribozymes. Here a weighting factor is a mutation-derived score normalized by the mean of mutation-derived scores. Mutation derived scores are from R-scape, Evolutionary couplings (EC-RNA), mean-field direct coupling analysis (mfDCA-RNA), CODA and epistasis. The same weighting factor was used by CODA+MC (2), R-scape (0.5) and EC-RNA (0.8) for both ribozymes. The weighting factor in mfDCA-RNA+MC is 1 for twister and 0.8 for CPEB3 for improving the performance of mfDCA-RNA+MC.


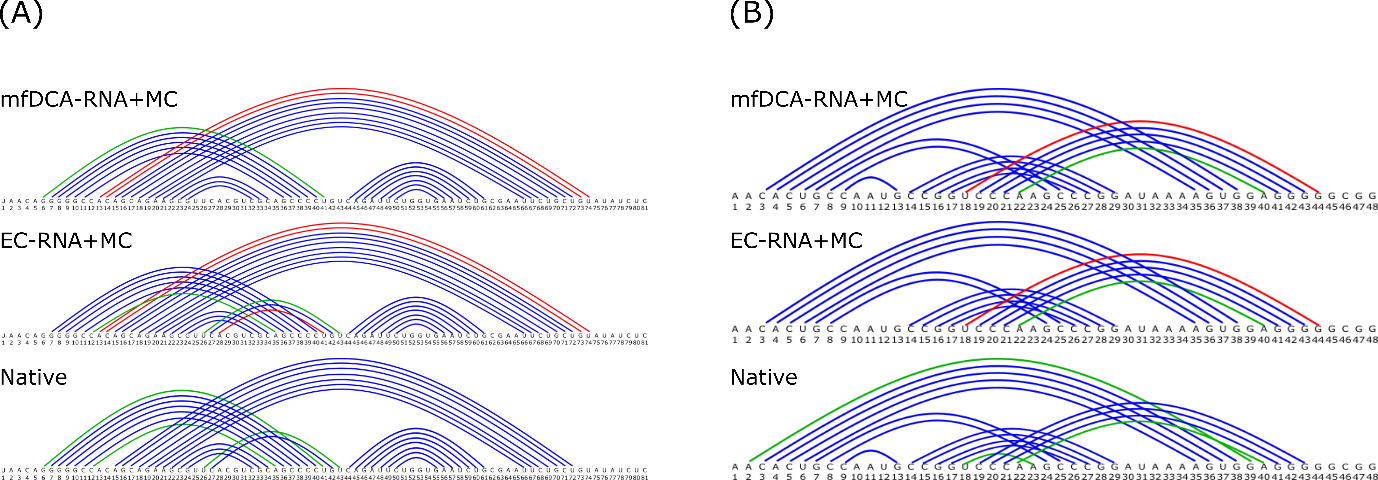


Figure S9. The base-pairing structures predicted by optimally combining mutation-derived scores with experimental base-pairing energies (mfDCA-RNA+MC and EC-RNA+MC), as compared to the native structure. (A) CPEB3 (B) twister ribozyme. Native Watson-Crick and noncanonical base pairs are shown in blue and green, respectively. False positive predictions are shown in red.


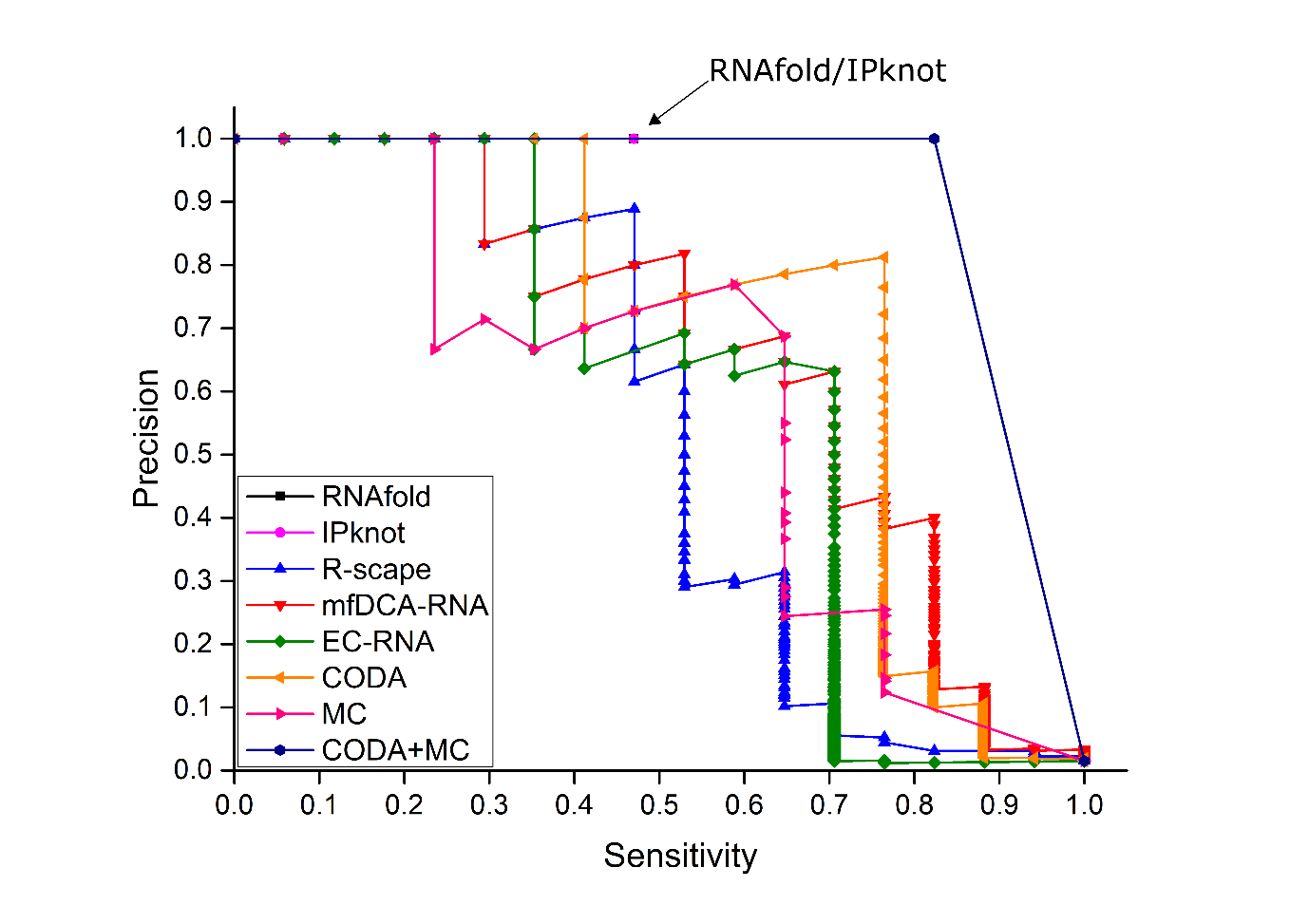


Figure S10. Precision (fraction of correct base pairs in predicted base pairs) versus sensitivity (coverage of known base pairs) by R-scape, mean-field direct coupling analysis (mfDCA-RNA), evolutionary couplings (EC-RNA), covariation-induced deviation of activity (CODA), Monte Carlo (MC) simulated annealing, and coupling of CODA and MC, using the deep mutation data from twister. Identical results from secondary structure predictor (RNAfold and IPknot) are also shown as a point (square and circle).


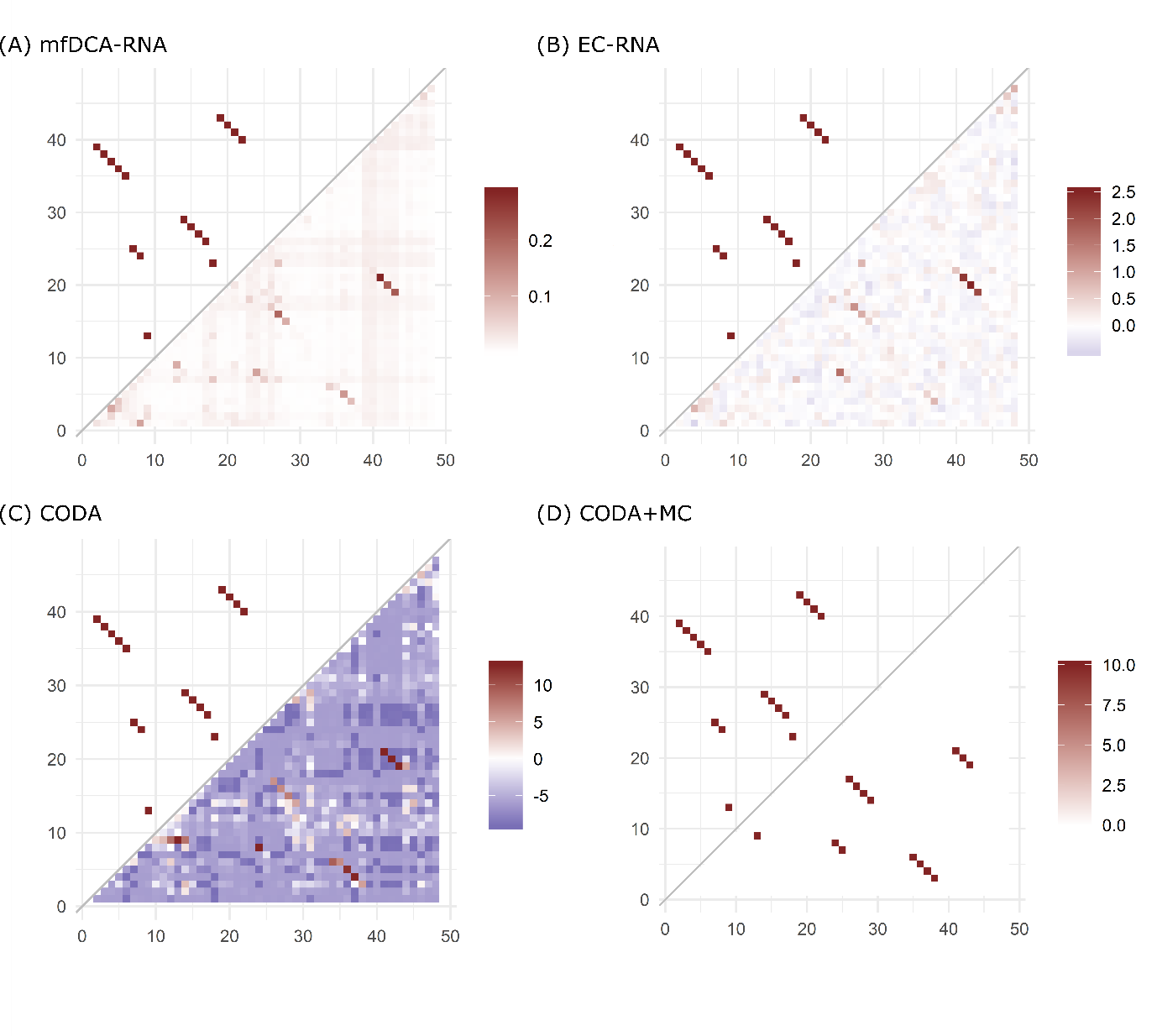


Figure S11. The comparison between native base-pairing map (upper triangle) of twister ribozyme and the map inferred from deep mutation data (lower triangle) of twister ribozyme by mfDCA-RNA, EC-RNA, CODA, and CODA+MC as labelled.


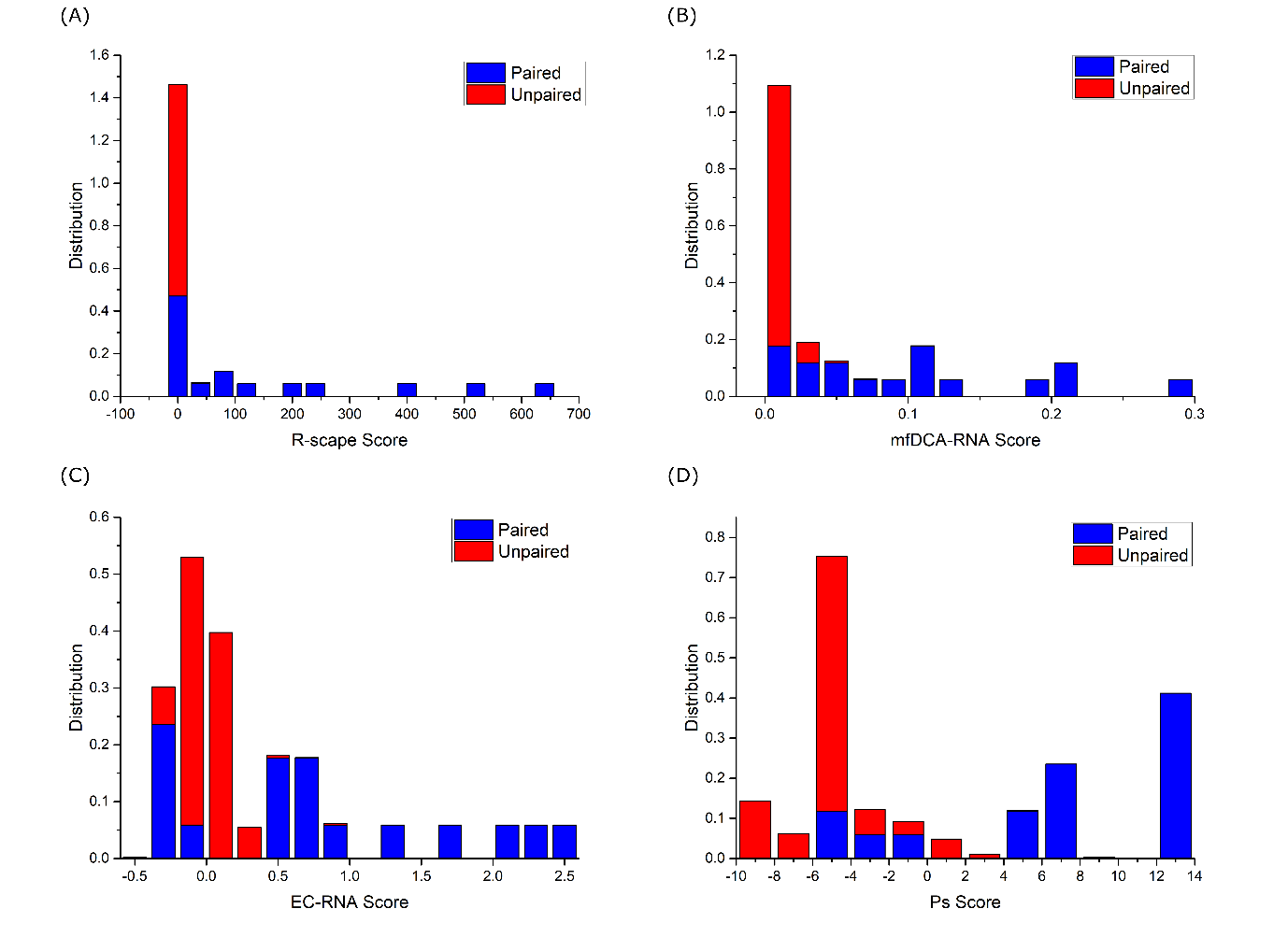


Figure S12. Distribution of R-scape (A), mfDCA-RNA (B), EC-RNA (C), and Paring Ps (CODA) (D) scores for bases paired and unpaired in twister ribozyme, respectively.


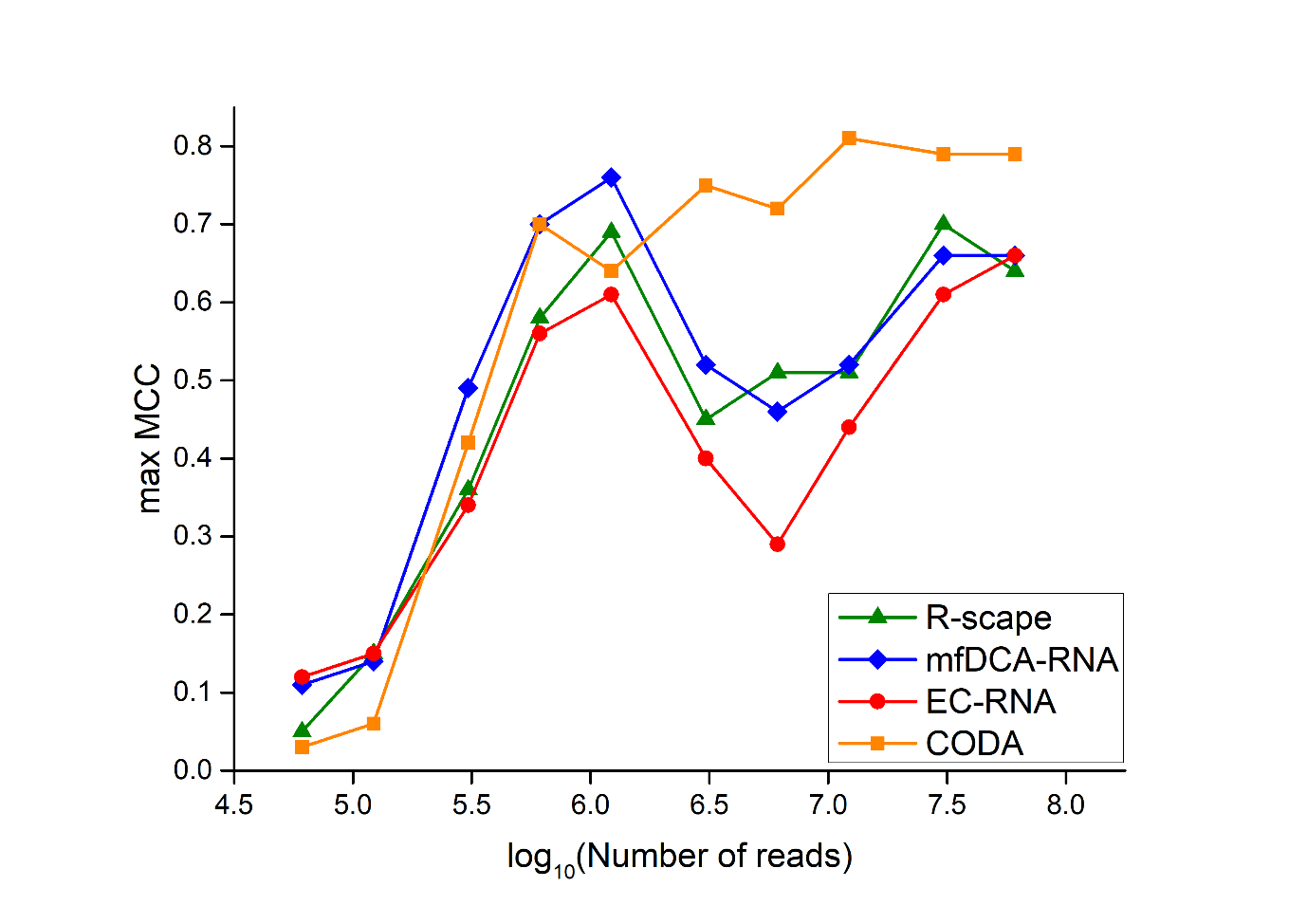


Figure S13. Matthews correlation coefficients by R-scape, mfDCA-RNA, EC-RNA and CODA as a function of the total number of reads for twister ribozyme by randomly removing the reads.


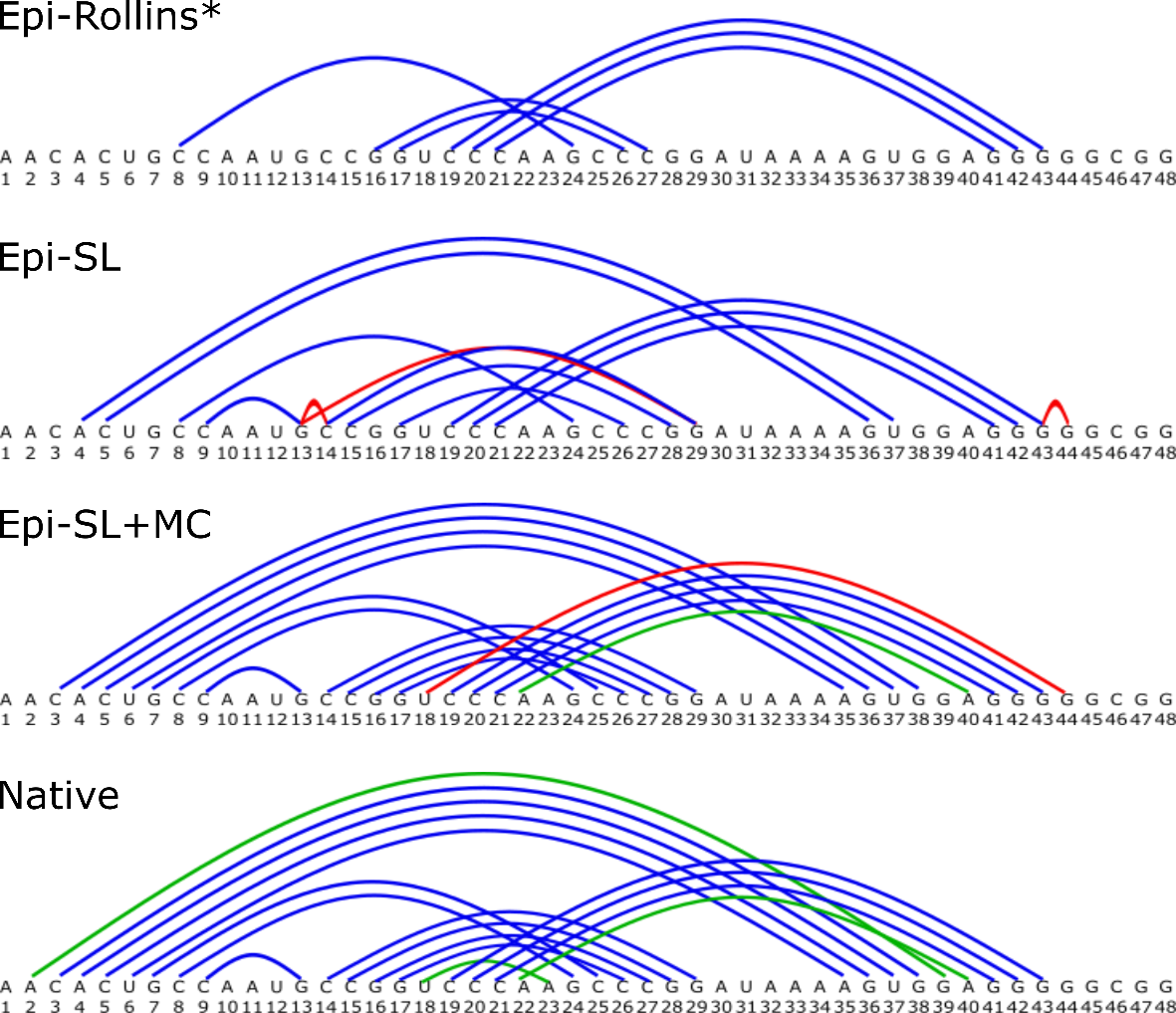


Figure S14. The base-pairing structure of the twister ribozyme inferred from its deep mutation data by epistasis analysis by Rollins et al. (Epi-Rollins), by Schmiedel and Lehner (Epi-SL) and by optimally combining Epi-SL with MC simulated annealing (Weighting factor of 0.8). Native Watson-Crick and noncanonical base pairs are shown in blue and green, respectively. False positive predictions are shown in red.
